# Stiffening Liquid Crystal Elastomers with Liquid Crystal Inclusions

**DOI:** 10.1002/adma.202504592

**Published:** 2025-06-09

**Authors:** Sahad Vasanji, Matthew Gene Scarfo, Arwa Alyami, Tizazu H. Mekonnen, Parsin Hajireza, Mohand O. Saed, Antal Jákli, Hamed Shahsavan

**Affiliations:** ^1^ Department of Chemical Engineering Institute for Polymer Research Center for Bioengineering and Biotechnology Waterloo Institute for Nanotechnology Waterloo ON N2L 3G1 Canada; ^2^ Department of Physics and Advanced Materials and Liquid Crystal Institute Kent State University Kent OH 44242 USA; ^3^ Department of Systems Design Engineering Center for Bioengineering and Biotechnology University of Waterloo Waterloo ON N2L 3G1 Canada; ^4^ Cavendish Laboratory University of Cambridge Cambridge CB3 0HE UK; ^5^ Present address: Department of Physics Najran University Najran Saudi Arabia

**Keywords:** cybotactic, induced smectic, liquid crystal elastomers, polymerization‐induced phase separation, soft actuators

## Abstract

Liquid crystal elastomers (LCEs) are promising building blocks for soft robots, given their large, programmable, reversible, and stimuli‐responsive shape change. Enhancing LCEs’ stiffness and toughness has been a longstanding desire previously explored by reinforcing them with fillers, crystalline microdomains, and interpenetrating polymer networks. While promising, these methods adversely affect molecular order and thermal strain. Here, a significant enhancement of the stiffness of LCEs is reported by loading them with low molecular weight liquid crystals (LMWLCs) without sacrificing thermal strain and molecular order. While pristine LCEs rapidly transition to a soft elastic plateau when strained from poly‐ to monodomain, LC‐loaded samples (LC‐LCEs) first experience a pronounced linear elasticity, followed by a soft elastic plateau at higher stresses. Further thermomechanical and X‐ray analysis confirm the emergence of an additional mesophase in polydomain LC‐LCEs, which evolves to short‐range smectic (cybotactic) during the poly‐ to monodomain transition. Monodomain LC‐LCEs show between 6.5‐ and 9.0‐fold stiffness enhancement with improved molecular order and thermal strain. Their work densities are more than double that of pristine LCEs, with active thermal stroke of up to 25% under loads of over 2000 times their weight. Such remarkable behaviors are attributed to the interplay between post‐polymerization phase separation of LCs and their strain‐enhanced smectic ordering. The results suggest that LMWLC inclusion can be a simple yet robust method to significantly improve the mechanical properties of LCEs.

## Graphical abstract

Incorporation of low molecular weight liquid crystals (LC) into liquid crystal elastomers (LCE) leads to a significant increase in their stiffness and output work density. Such remarkable stiffening is attributed to nanoscale phase‐separation and the formation of induced‐smectic domains in polydomain and monodomain LC‐LCEs, respectively.

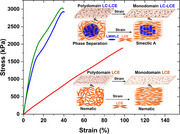

## 1 Introduction

Soft robots aim to recapitulate the functionality of traditional robots by replacing rigid motors and pumps with soft organic artificial muscles. They offer advantages over their rigid‐bodied cousins in technologies where safe human interactions, boundless degrees of freedom in motion, large strains, and lightness are required.^[^ ^1^, ^2^, ^3^ ^]^ Among a variety of artificial muscles, liquid crystal elastomers (LCEs) have attracted much attention in the past decade. LCEs are polymers containing liquid crystals (LCs), either in their backbone or pendant to them, loosely cross‐linked to elastic networks. Enjoying both elasticity and liquid crystalline order, LCEs exhibit large, reversible, anisotropic, and programmable deformations in response to various stimuli.^[^ ^4^, ^5^, ^6^ ^]^ However, soft artificial muscles made from pure LCEs typically suffer from low output work density, which must be enhanced by improving their mechanical properties, including stiffness and toughness.

To this end, higher cross‐linking, and the inclusion of crystalline microdomains, interpenetrating polymer networks, and fillers into LCEs have been previously explored. While high content of cross‐linking leads to enhanced stiffness of LCEs, it drastically reduces toughness and thermal strain.^[^ ^7^ ^]^ In a different approach, Kim and coworkers showed that LCEs made from LC mesogens oligomerized with long aliphatic chain extenders crystallize over time, adapting thermomechanical properties similar to semicrystalline polymers (i.e., melting), and develop large stiffness, toughness, and thermal strain actuation.^[^ ^7^ ^]^ Lu and coworkers demonstrated that interpenetrating networks of main‐chain polyurethanes and LC polyacrylate thermosets have significantly large stiffness, toughness, thermal strain, and work capacity.^[^ ^8^ ^]^

Loading LCEs with organic solid particles, such as carbon black, nanotubes, graphene, and cellulose, and inorganic ones, such as metals, metal oxides, and ceramics, is perhaps a more facile and common strategy to modify LCEs’ mechanical properties. The literature is rich with LCE composites, which gained not only mechanical reinforcement but also other functionalities, including photothermal response and electrical/magnetic activity, thanks to the inclusion of solid particles. However, this method typically comes at the cost of their extensibility, thermal strain, LC order, and significantly altered phasic behavior.^[^ ^9^, ^10^ ^]^ In contrast to solid particle‐LCE composites, Ford and coworkers recently dispersed droplets of liquid metals (LM), including Eutectic Indium‐Gallium (EGaIn) and pure Gallium (Ga), into a thiol‐acrylate LCE matrix (LM‐LCE). They showed that the inclusion of LM droplets of sizes ranging from 200 to 500 µm into polydomain LCEs does not adversely affect extensibility and elastic modulus (calculated beyond the soft plateau). Their results, however, indicate a notable difference between the strain‐stress behavior of LM‐LCEs and pristine LCEs. Upon stretching, their pristine polydomain LCE rapidly transitions to a soft elastic plateau, while LM‐LCEs initially experience a pronounced linear elasticity before such a transition.^[^ ^11^ ^]^ In a sequel work, they showed that reducing the liquid EGaIn droplet size from ≈100 to ≈1 µm significantly increases their elastic modulus (>10 folds), and LM‐LCEs with smaller LM inclusions have marked linear elasticity upon stretching.^[^ ^12^, ^13^ ^]^ In a separate work, Lu et al. decreased the size of LM droplets in their LM‐LCEs even further, 195 nm, and enhanced the elastic modulus >2 folds.^[^ ^14^ ^]^ These results suggest that inclusion of liquids into LCE solids can improve their stiffness, particularly for systems with much smaller inclusions. However, stiffening in these reports caused a large reduction in LCEs’ extensibility, molecular order, phase transition, and thermal strain.

Interestingly, stiffening LCEs with LM inclusions (droplets of <10 µm) appears to be in line with Style and coworkers’ observation on “stiffening solids with liquid inclusions.”^[^ ^15^, ^16^ ^]^ In their seminal works, they showed that Eshelby's inclusion theory breaks down for liquid‐solid composites if the radius of liquid inclusions (*R*) is smaller than the elastocapillary length *L*≅γ/*E*, where γ is the surface tension and *E* is the elastic modulus of the solid. When *R* ≤ *L*, the surface energy of liquid inclusions competes with applied strain energy, resisting the deformation of the liquid inclusions and increasing the macroscopic stiffness of liquid‐solid composites. Therefore, capillary effects must be accounted for in predicting far‐field stress‐strain behavior of elastomers, with a typical modulus on the order of megapascals, at scales on the order of tens and hundreds of nanometers. Note that Style and coworkers’ results are based on the inclusion of *isotropic* (non‐liquid crystalline) liquids into *isotropic* (non‐liquid crystalline) soft solids.^[^ ^15^, ^16^ ^]^ Ford and coworkers’ and Lu and coworkers’ results are based on the inclusion of *isotropic* liquids (Ga and EGaIn) into *anisotropic* soft solids (LCEs). To the best of our knowledge, the impact of the inclusion of *anisotropic* liquids, like nonreactive low molecular weight LCs (LMWLCs), on the far‐field stress‐strain, molecular order, and thermal strain actuation of thiol‐acrylate LCEs has not been explored systematically.

Here, we demonstrate that incorporating common LMWLCs, such as 4‐Cyano‐4'‐pentylbiphenyl (5CB) and 4‐Cyano‐4'‐octylbiphenyl (8CB), into thiol‐acrylate LCEs significantly affects their phase behavior, molecular order, and thermomechanical properties. We observe that loading the precursor of LCEs with specific amounts of 5CB and 8CB (denoted as LC‐LCEs) induces phase separation within polydomain LC‐LCEs obtained by thiol‐Michael addition (first stage of cross‐linking) and substantially alters their thermomechanical properties. The transition from poly‐ to monodomain in LC‐LCEs during stretching enhances the short‐range smectic ordering within LC‐LCEs, reminiscent of cybotactic systems. Freezing in such smectic ordering in monodomain LC‐LCEs via photopolymerization (second stage of cross‐linking) results in a significant increase in Young's modulus. Monodomain LC‐LCEs loaded by 5CB and 8CB show 650% and 900% enhancement in Young's modulus compared to their pristine LCE counterpart, respectively. Additionally, work densities of monodomain LC‐LCEs calculated from active thermal stroke are over twice as much as the control LCE. Unlike earlier reports on the inclusion of liquid metals in LCEs, the notable enhancement in mechanical properties observed here does not compromise molecular order, phase transitions, or thermal strain. Our findings indicate that the inclusion of liquid crystals can serve as a straightforward yet effective method for significantly boosting the mechanical properties of thiol‐acrylate LCEs.

## 2 Results and Discussion

### 2.1 Post‐Polymerization Phase Separation

Nonreactive LMWLCs, such as 5CB, mixed with reactive liquid crystalline acrylates, such as RM257, undergo phase separation after photo‐polymerization despite their miscibility.^[^ ^17^, ^18^, ^19^, ^20^, ^21^ ^]^ Below a certain concentration, unbounded LMWLCs follow, or slightly alter, the phase behavior of the polymerized network surrounding them and act merely as plasticizers.^[^ ^22^ ^]^ Above a certain concentration, however, unbounded LMWLCs may form LC‐rich domains that alter their phase behavior, such as melting point (*T_m_*) or nematic‐isotropic transition temperature (*T_NI_*).^[^ ^17^, ^23^ ^]^ In addition, polymerization‐induced phase separation of LMWLC inclusions from cross‐linked networks of reactive mesogens influences their microstructure and morphology, which can be leveraged for the shape‐change programming of LC‐loaded networks.^[^ ^20^, ^21^ ^]^ Morphological micrographs of LC‐loaded cross‐linked acrylate networks available in the literature suggest that one can architect the shape and size of the phase‐separated domains by changing the content of non‐reactive LMWLCs, the chemistry of cross‐linked matrix, and the miscibility between the LCs and cross‐linked matrices.^[^ ^20^, ^21^ ^]^ We postulate that the LC‐LCEs’ stiffness, too, can be tailored by changing the shape, size, and morphology of the LC‐rich phase‐separated domains.

**Figure 1:**
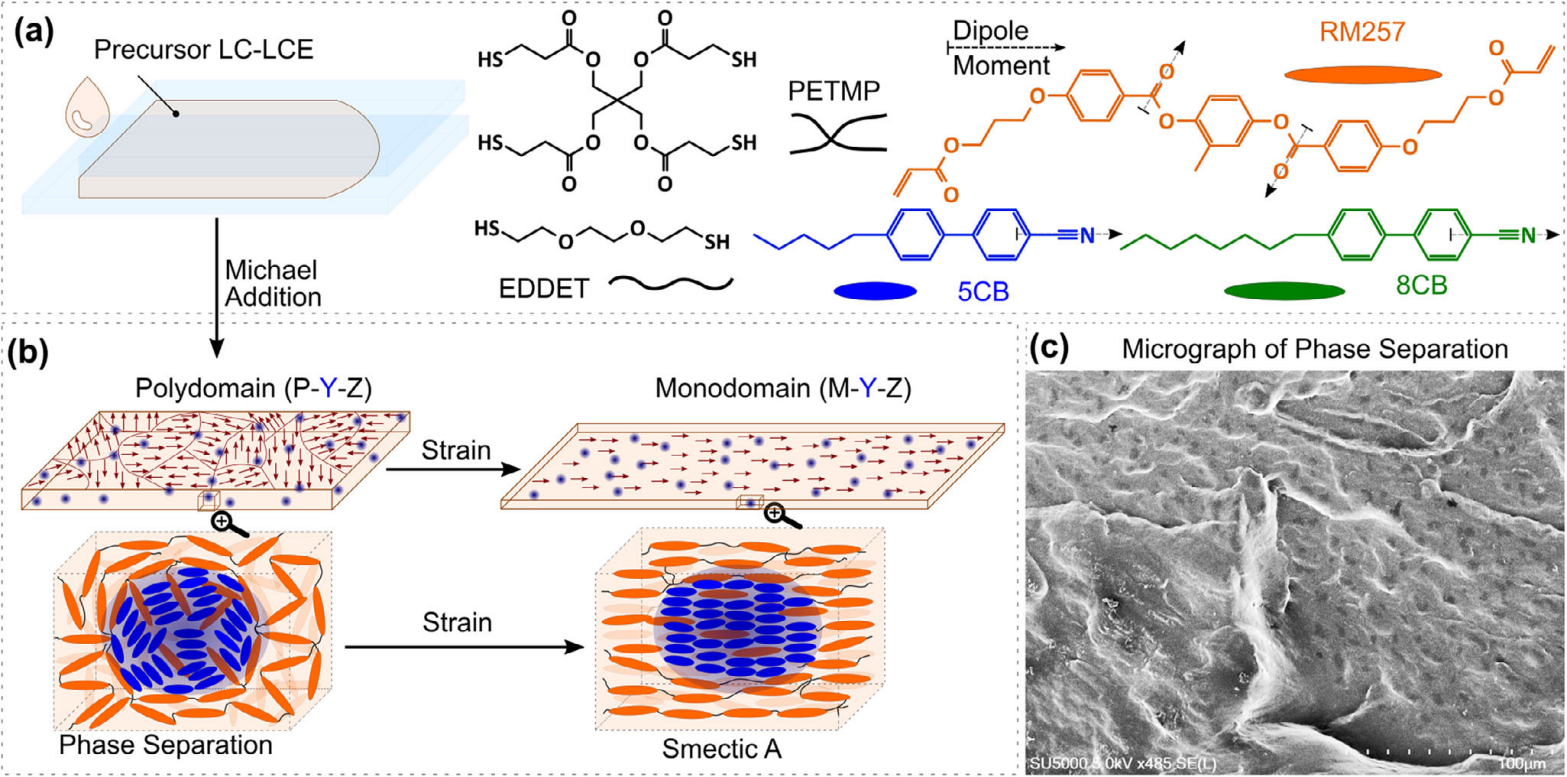
LCE chemistry and molecular ordering. a) Chemical structures of thiol cross‐linker and chain extender (black), nematic LC monomer (orange), and unreactive nematic (blue) and smectic (green) mesogens. b) In the polydomain LCE after the first stage polymerization, LMWLCs phase separate from the matrix. LMWLC‐rich domains, phase‐separated within polydomain LC‐LCEs, may contain randomly oriented smectic clusters that align during the polydomain to monodomain transition. c) SEM micrograph of P‐5‐70 containing features resembling phase‐separated domains (dark‐grey dimples).

To test our hypothesis, we mixed different amounts of 5CB (Cr 24 °C N 35.5 °C I) and 8CB (Cr 21.5 °C SmA 33 °C N 40.5 °C I) with a typical two‐stage thiol‐acrylate LCE precursor that contains a diacrylate nematic mesogen (RM257) combined in excess with a tetra‐thiol crosslinker (Pentaerythritol tetrakis(3‐mercapto propionate): PETMP) and a dithiol chain extender (2,2’‐(Ethylenedioxy) ethanethiol: EDDET) (**Figure** **1**a).^[^ ^24^ ^]^ For brevity, we refer to LC‐LCEs as “X‐Y‐Z,” where X is either P or M, representing poly‐ or monodomain samples, respectively; Y is 5 or 8 for 5CB or 8CB; and Z indicates the additional weight percentage of 5CB/8CB used in the LC‐LCE formulations, ranging from 10% wt. to 70% wt. of the original formulation. Control poly‐ and monodomain LCEs without LC inclusions are labeled as P0 and M0, respectively. We hypothesized that LMWLCs would form phase‐separated domains after the first stage of polymerization/cross‐linking and undergo polydomain to monodomain transition during a subsequent uniaxial alignment before photo‐polymerization in the second stage (Figure 1b,c). Considering the high miscibility measured and calculated between 5CB and LCE (for further details, see Note S1, Supporting Information), we anticipated relatively small phase‐separated domains in the micron to nanometer range. Examination of etched and cryo‐fractured P‐5‐Z specimens using scanning electron microscopy (SEM) confirmed the presence of tiny dimples (<10 µ*m*). However, we could observe such morphology only for P‐5‐70 (Figure 1d) and could not visually confirm the existence of such dimples in the SEM micrographs of LC‐LCEs with smaller amounts of 5CB. It is tempting to attribute the observed dimples to phase separation, but we cannot confidently do so, given the lack of evidence from SEM micrographs of other formulations.

**Figure 2:**
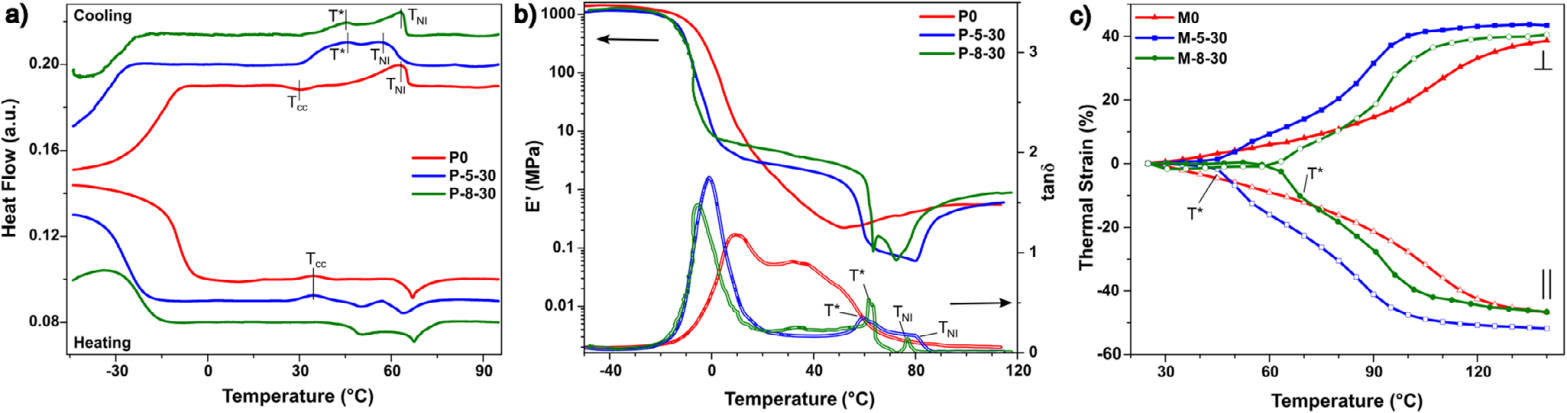
LCE Thermophysical properties. a) DSC heat flow *(exo‐up)* against temperature for polydomain samples with T_NI_ and additional mesophase transition T* labeled. The top graphs show the first cooling cycle, and the bottom ones show the second heating cycle. Vertical dashed line T_cc_ shows cold crystallization. b) Top graphs (solid lines arrowed to the left) show *E'* and bottom graphs (hollow lines arrowed to the right) show *tanδ* for polydomain samples. c) Thermal strain for monodomain samples with positive (negative) representing strains perpendicular (parallel) to the director.

To explore the impact of 5CB inclusion on the phasic behavior of LC‐LCEs, we conducted a thermal and thermomechanical analysis of P‐5‐Z samples using Differential Scanning Calorimetry (DSC) and Dynamic Mechanical Analysis (DMA). DSC data in Figure S2 (Supporting Information) shows a general decreasing trend for the *T_g_* as a result of LC inclusion. Moreover, the first cooling data shows an additional peak below the $T_{NI}^{P-5-Z}$ closer to the $T_{NI}^{5CB}$, especially for P‐5‐50 (T* ≈ 37 °C) and P‐5‐70 (T* ≈ 36 °C). This behavior is attributed to the coexistence of two separate populations of 5CB molecules: one population being mobile, manifested by a phase transition around $T_{NI}^{5CB}$ and the other bound to the LCE, plasticizing it and reducing the $T_{NI}^{P0}$.^[^ ^17^ ^]^ The additional peak on the DSC cooling data of P‐5‐30 is farther from $T_{NI}^{5CB}$ and overlaps with the $T_{NI}^{P-5-30}$ (T* ≈ 45 °C), also shown in **Figure** **2**a, with a notably larger enthalpy than that of P‐5‐50 and P‐5‐70.

We also conducted a DMA analysis of P‐5‐Z samples to cross‐examine the impact of 5CB inclusion on the phasic behavior of LCEs. Variation of storage modulus (*E*′) and loss factor (*tan* δ) with temperature (Figure S3, Supporting Information) shows that the inclusion of 5CB systematically reduces the *T_g_* of LCE. P0 somehow displays previously documented characteristics of polydomain LCEs, including a heightened *tan* δ plateau between $T_{g}^{P0}$ and $T_{NI}^{P0}$, followed by its decline approaching the clearing point.^[^ ^25^ ^]^ Stark differences between P0 and P‐5‐Z are observed with the emergence of an intermediary transition between the *T_g_* and *T_NI_* for P‐5‐30, P‐5‐50, and P‐5‐70 storage moduli, and a peak (T*) before *T_NI_* on their *tan* δ. The loss factor for P‐5‐50 and P‐5‐70 became extremely noisy, overshadowing the transitions at higher temperatures, which can be attributed to the large content of 5CB and thermal softening beyond T* (Figure S3c, Supporting Information).

Interestingly, DSC and DMA graphs containing the additional mesophase resemble those of LCEs possessing both smectic and nematic mesophases, even though both thiol‐acrylate LCE matrix and 5CB dopant typically show nematic mesophase.^[^ ^26^, ^27^ ^]^ To gain further insight, we conducted DSC (Figure 2a) and DMA (Figure 2b) experiments with identical protocols this time on P‐8‐30 samples. To compare the effect of 8CB's phase behavior on phase‐separated domains, we chose to test only one formulation with 30 wt.% of 8CB, P‐8‐30, and compared it to its analog, P‐5‐30. 8CB shows smectic mesophase at room temperature and we assume that phase‐separated domains in P‐8‐30 could also adapt smectic ordering. Our results show that the inclusion of 8CB, too, lowers *T_g_* by endowing the LCE with larger chain mobility at lower temperatures. Unlike 5CB, 8CB elevates the $T_{NI}^{P0}$, which can be attributed to a larger energy requirement for disrupting the increased intermolecular interactions stemming from the dimeric nature of 8CB.^[^ ^28^ ^]^ DSC data on the first cooling show that P‐8‐30 has a more distinct peak (T* ≈ 46 °C), albeit with a lower transition enthalpy than that of P‐5‐30. Likewise, there is a significant difference between the storage modulus of P0 and P‐8‐30 (Figure 2b). The intermediary transition peak on the *tan* δ data of P‐8‐30 is sharper than that of P‐5‐30. The large (+ 5 °C) difference between T* and $T_{NI}^{8CB}$ can be attributed to the strong interaction of cyanobiphenyl molecules with the surrounding network.^[^ ^17^ ^]^

To examine the dependency of the additional mesophase on the degree of alignment, we measured the thermal strain (ɛ_*T*_ = Δ*l*/*l*_0_ ) of monodomain specimens. For this, we applied uniaxial strain on P0, P‐5‐30, and P‐8‐30, and then proceeded with the second stage of cross‐linking by exposing the strained samples to ultraviolet (UV) light to obtain monodomain samples (M0, M‐5‐30, and M‐8‐30). The strain at which each formulation is deemed to be transitioned from polydomain to monodomain was calculated by the real‐time measurement of the transmission coefficient against the strain, following a previously reported protocol (Note S2, Supporting Information).^[^ ^29^ ^]^ As can be seen in Figure 2c, ɛ_*T*_ for M0 monotonically decreases (increases) with temperature parallel (perpendicular) to the alignment. On the contrary, ɛ_*T*_ for M‐5‐30 displays notable slope changes at the proximity of T* measured in DSC and before *T_NI_*. For M‐8‐30, the slope change takes place at higher temperatures than the measured T* in DSC. For all samples, the ɛ_*T*_ change associated with the *T_NI_* occurs at substantially larger temperatures. This is probably due to the supercritical behavior of mechanically strained and cross‐linked monodomain samples, which masks the first‐order nematic‐isotropic transition and favors a nematic‐paranematic transition requiring more heat to disrupt the mesophase order.^[^ ^30^, ^31^ ^]^ Taken together, these three experiments confirm the creation of a new mesophase upon the inclusion of LCs in LCEs, regardless of LCs’ phasic signature.

### 2.2 Evidence of Cybotactic Order

**Figure 3:**
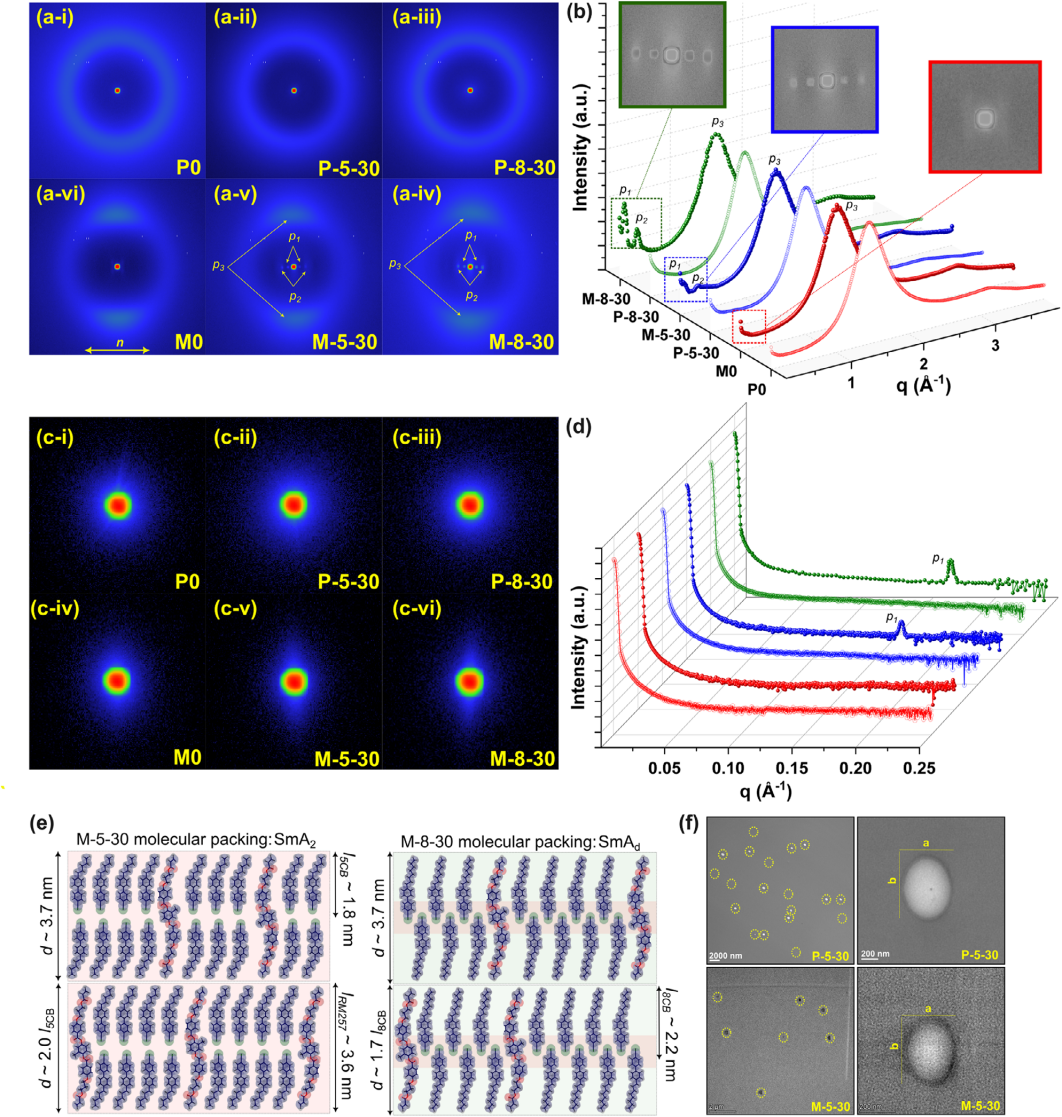
Characterization of molecular ordering and nanostructure. a) WAXS 2D scattering patterns, and b) radially integrated 1D scattering plots for P0, P‐5‐30, P‐8‐30, M0, M‐5‐30, and M‐8‐30. The horizontal yellow arrow indicates the stretch direction for all samples. Peaks resulting from spatial periodicity are labeled *p_i_*. Inset figures in (b) show magnified and color‐enhanced small‐angle regions with distinct *p_1_* and *p_2_* for LC‐LCEs. c) SAXS 2D scattering patterns and d) radially integrated 1D scattering plot for P0, P‐5‐30, P‐8‐30, M0, M‐5‐30, and M‐8‐30. e) proposed scenario for molecular packing within LMWLC‐rich phase‐separated domains. f) TEM micrograph of P‐5‐30 and M‐5‐30 showing the phase‐separated domains within dashed yellow circles and a magnified domain with a characteristic radius of (a + b)/4.

We conducted Small‐Angle X‐ray Scattering (SAXS) and Wide‐Angle X‐ray Scattering (WAXS) studies to unravel the nature of the additional mesophase observed in the previous experiments and determine the molecular packing within phase‐separated domains. The equipment covered scattering vectors (*q*) between 0.001 and 0.2 Å^−1^ in SAXS and between 0.1 and 4.0 Å^−1^ in the WAXS modes, respectively, with an overlap between 0.1 and 0.2 Å^−1^. **Figure** **3**a (i–iii) and Figure 3b show the 2D WAXS scattering patterns and integrated 1D profile (scattering intensity *I* vs scattering vector *q*) of P0, P‐5‐30, and P‐8‐30 obtained at 25 °C, respectively. 2D WAXS scattering patterns of all samples show a diffuse ring at wide angles (corresponding to an equatorial peak *p_3_* around ca. 1.4 Å^−1^), which can be attributed to the lack of orientational order in nematic polydomain samples.^[^ ^32^ ^]^ Such a lack of orientational order in P0, P‐5‐30, and P‐8‐30 was also observed in SAXS results, where 1D profiles did not have any distinct peaks, and 2D scattering patterns were isotropic (Figure 3c(i–iii),d). SAXS scattering intensities of P‐5‐30 and P‐8‐30 being larger than that of P0 at the same scattering vectors can be attributed to nanophase separation into LMWLC‐rich and LCE‐rich domains.

Upon stretching, equatorial rings, shown in Figure 3a(i–iii), collapsed into diffuse arches (Figure 3a(iv–vi)), indicative of the formation of an orientational order and a global director *n*. WAXS scattering patterns of P‐5‐30 and P‐8‐30, which were stretched by 50%, started to develop subtle pseudo‐Bragg peaks in the small‐angle region, reminiscent of smectic‐A ordering (SmA) (Figure S5, Supporting Information). These peaks intensify and morph during a full transition from poly to monodomain. With ≈235% and 194% stretching, M‐5‐30 and M‐8‐30 develop two distinct dots (corresponding to meridional peaks *p_1_* and *p_2_*) representing condensed pseudo‐Bragg peaks. In Figure 3b, we can also see that the 1D WAXS profiles of M‐5‐30 and M‐8‐30 have two peaks within the small‐angle region labeled as *p_1_* (ca. 0.175 Å^−1^) and *p_2_* (ca. 0.35 Å^−1^), in addition to *p_3_* present in all samples. The presence of an orientational order within M0, M‐5‐30, and M‐8‐30 can also be confirmed in SAXS results, where 2D scattering patterns morph into anisotropic ovals (Figure 3c(iv–vi)), and 1D profiles develop a peak corresponding to *p_1_* (ca. 0.175 Å^−1^). This peak intensifies with strain (Figure S5, Supporting Information; Figure 3d). The order parameter (*S*) of P‐5‐30 and P‐8‐30 is much smaller than that of P0, but it substantially increases upon stretching (Note S3 and Table S3, Supporting Information). These results suggest that the strain not only induces orientational alignment but also the formation or enhancement of SmA‐like layers perpendicular to the director and stress field.^[^ ^17^, ^32^, ^33^ ^]^ We speculate that far‐field stress and strain during the alignment process facilitate the proximity and enhance alignment of 5CB and 8CB molecules within LMWLC‐rich domains. This leads to or intensifies the charge transfer between aromatic rings (electron donors) and cyano groups (electron acceptors) within these domains, and consequently, the evolution of uniform smectic ordering.^[^ ^34^ ^]^ Attenuated Total Reflectance Fourier Transfer Infrared Spectroscopy (ATR‐FTIR) measurements before and after stretching, indeed, show a shift in cyano‐group absorption frequency (ν_*CN*_) during P‐5‐30 → M‐5‐30 (1.63 cm^−1^) and P‐8‐30 → M‐8‐30 (3.61 cm^−1^) transitions (Note S4 and Figure S6, Supporting Information).^[^ ^35^, ^36^, ^37^ ^]^

To obtain insight into the molecular packing within the phase‐separated domains, we baseline‐corrected 1D WAXS and 1D SAXS profiles of M‐5‐30 and M‐8‐30, and calculated layer spacings (*d*_*i*,*x*_ = 2π/*q* ), and correlation length (ξ_*i*,*x*_ = 2*K*π/*FWHM* ) corresponding to *p_1_*, *p_2_*, and *p_3_* (Table S4, Supporting Information). In these equations, *i* is the peak number, *x* is the name of the sample, *K* is the shape factor, which is approximated as 1 for simplicity,^[^ ^38^ ^]^ and FWHM is the full‐width at half‐maximum. For all monodomain samples *d*_3,*x*_ ≈  4.5 Å, which correlates with the width of aromatic rings in mesogens and the intermolecular distance in the equatorial direction (i.e., perpendicular to *n*). Meridional peaks (*p_1_* and *p_2_*) arise from longitudinal intermolecular distances and describe the smectic‐like layer spacing *d*_1,*x*_.^[^ ^39^ ^]^ From 1D WAXS profiles, we estimated the layer spacing corresponding to *p_1_* and *p_2_* in M‐5‐30 and M‐8‐30 to be ≈37 and ≈18 Å, respectively. Moreover, from the SAXS 1D profile, we could extract layer spacing corresponding to *p_1_* for both M‐5‐30 and M‐8‐30 samples, both turned out to be ≈37 Å.

The formation of SmA‐like structures between molecules of higher homologs of 4‐Cyano‐4‐alkylbiphenyl is well‐documented and attributed to the charge transfer or dipole‐dipole interactions between them. It is known that dimers of antiparallel cyanobiphenyl molecules stack into layers with a thickness (*d*) between 1 and 2 times the molecular length (*l*). The longer the cyanobiphenyl molecule, the larger the longitudinal dipole and the more stable its smectic mesophase.^[^ ^40^, ^41^ ^]^ For cyanobiphenyls, the literature suggests that *d*/*l* is typically ≈1.4, which is the fingerprint of a so‐called SmA_d_ mesophase (Figure 3e). However, in longer polar (cyano‐ or nitro‐terminated) LCs with lateral dipoles, such as molecules with core ester groups joining aromatic rings, the electron density distribution along the long axis of the molecules changes and other polymorphic mesophases, such as SmA_2_, with *d*/*l*  ≈  2 (Figure 3e) could be observed.^[^ ^42^, ^43^, ^44^, ^45^ ^]^

In one likely scenario, having twice the *q*‐value of *p_1_*, *p_2_* could be considered as a second order reflection with smectic structuring.^[^ ^46^, ^47^ ^]^ *p_1_* will be the main and the first‐order reflection meridional peak with layer spacing (≈37 Å), which is slightly larger than 2 × *l*_5*CB*_ (≈18 Å)^[^ ^48^ ^]^ and 1.67 × *l*_8*CB*_ (≈22.1 Å).^[^ ^49^ ^]^ Therefore, the molecular packing within the phase‐separated domains of M‐5‐30 and M‐8‐30 most likely resembles that of SmA_2_ and SmA_d_, respectively. RM257 (*l*_*RM*257_ ≈  36 Å) could also fit within each smectic layer. Note that the deviation of layer spacing of 8CB and 5CB dimers from the commonly observed 1.4*l* may be attributed to the impact of lateral dipoles of RM257 molecules, particularly in the vicinity of their carbonyl groups. As such, we speculate that RM257 molecules are present and solvated within LMWLC‐rich domains. This scenario, which is depicted in Figure 3e, is similar to the formation of smectic A clusters from phase separation of 8CB and E7 within RM257 after photopolymerization.^[^ ^17^, ^50^ ^]^ Following this scenario, we believe that reduced electron density transfer to cyano‐groups in P‐5‐30 → M‐5‐30 transition may cause less dense molecular packing within the layers of 5CB (longer and looser dimers) as well as higher free volume between layers (larger spacing) compared to that of 8CB (P‐8‐30 → M‐8‐30).^[^ ^34^ ^]^ In another scenario, one could argue that *p_1_* and *p_2_* are not first and second‐order reflections and each could represent a certain population of molecules with their respective smectic ordering.^[^ ^32^ ^]^ However, *d*_2,*M* − 8 − 30_ ≈ 18 Å is incompatible with the layer spacing being equivalent to the length of 8CB or their dimers, and hence, this scenario is unlikely.^[^ ^41^, ^50^, ^51^ ^]^

Performing small‐angle neutron scattering is key to accurately determining the size of separated smectic domains, but, unfortunately, it was not available at the time of the experiments. In a crude approximation, we can assume these domains are larger than 10 nm, which is the correlation length of liquid‐crystalline phase formation, close to ξ_3,*x*_ shown in Table S4 (Supporting Information). For samples with 30 wt.% 5CB or 8CB, the size of these domains cannot be greater than a micron because we cannot see them under a microscope.^[^ ^27^ ^]^ In a more accurate approximation, we calculated ξ_1,*x*_ from the peak observed on both SAXS data and WAXS, which may provide an upper limit on the size of the phase‐separated domains in monodomain samples. The discrepancy between ξ_1,*M* − 5 − 30_, and ξ_1,*M* − 8 − 30_ obtained from SAXS and WAXS data (220 nm vs 9.3 nm for M‐5‐30 and 210 nm vs 12.2 nm for M‐8‐30) can be rooted in the fact that WAXS peaks are not well‐isolated and may have overlap with other peaks (harmonics), which could be analyzed only through secondary data processing (Note S5 and Figures S7–S9, Supporting Information). Although SAXS has higher resolutions in the acquisition of small‐angle data, the q‐value for *p_1_* is on the higher end of the method's range, where the data starts to become noisier. To be conservative, we favor the data obtained from SAXS as the upper limit for the calculation of correlation length and speculate that the correlation lengths of M‐5‐30 and M‐8‐30 are ≈220 nm and 210 nm, respectively. Interestingly, our further examination of P‐5‐30 and M‐5‐30 with transmission electron microscopy (TEM) confirmed the presence of phase‐separated domains with an average characteristic inclusion radius, *R* ≈ (*a* + *b*)/4, of 156 ± 22 and 130 ± 22 nm, respectively (Figure 3f). Further scrutiny into the molecular packing within LMWLC‐rich phase‐separated domains was not viable as the contrast between the domains and surrounding LCE began to fade, followed by burning of the samples whenever the beam became stronger. Altogether, we believe that our LC‐LCEs display nematic cybotactic ordering comprised of uniaxially aligned short‐range SmA_d_ or SmA_2_ domains for M‐8‐30 and M‐5‐30, respectively.^[^ ^48^, ^52^, ^53^ ^]^

### 2.3 Mechanical Properties of LC‐LCEs

**Figure 4:**
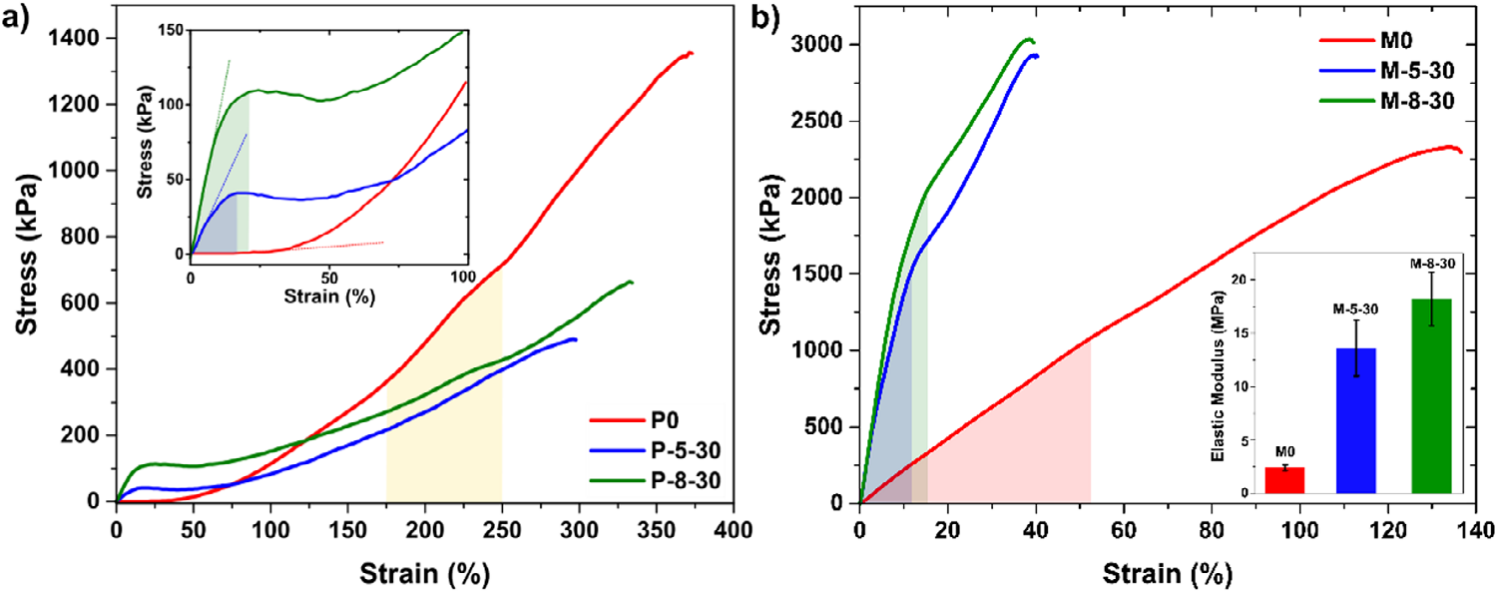
Mechanical properties. a) Stress‐strain curves for polydomain samples with the inset showing the magnified low‐strain behavior. Areas shaded with green and blue show the pre‐soft‐elastic plateau region where we measured initial elastic moduli. We calculated elastic moduli post‐soft‐elastic plateau from the slope of the curves within the area shaded with yellow. Note: samples were tested on day 5 post‐synthesis. b) Monodomain tensile stress‐strain curves parallel to the director. The elastic moduli shown in the inset are calculated from the slope of the graphs in the shaded areas.

**Figure** **4**a shows the mechanical properties of P0, P‐5‐30, and P‐8‐30. Like other reports on thiol‐acrylate LCEs^[^ ^31^ ^]^ P0 rapidly transitions from a linear elastic region upon stretching (ɛ <  5%). Elastic modulus of P0 calculated just before the soft‐elastic plateau was <30 kPa. Note that some of our P0 samples did not demonstrate an initial linear elastic region, due to the procedure we took for their fabrication. All our samples were fabricated inside glass capillary cells, and after the first stage of cross‐linking, were peeled off the glass substrates. We believe that the peeling process caused many of our samples to surpass their initial linear elastic region before they were tested in the tensile setup. But the procedure is common for all LCE and LC‐LCE samples and would not affect our argument. Polydomain to monodomain transition for P0 starts with an extended soft‐elastic plateau (ɛ <  50%), followed by a linear elastic and strain‐hardening behavior after mesogens align parallel to the far‐field stress direction. Elastic modulus of P0 calculated at 185% < ɛ <  235% was ≈490 kPa. The elongation and toughness at break of P0 were ≈370%, and 2035 kJ m^−3^.

In contrast, both P‐5‐30 and P‐8‐30 demonstrate a distinct linear elastic behavior (ɛ < 12.5%) with elastic moduli calculated at ≈413 and ≈970 kPa, respectively. A similar emergence of pronounced linear elastic behavior and stiffening was also observed for thiol‐acrylate LCEs doped with liquid metals.^[^ ^11^, ^12^ ^]^ Note that in our studies, the initial linear elastic behavior observed for polydomain LC‐LCEs evolved with time and peaked after 5 days post‐synthesis (Note S6 and Figure S10, Supporting Information). Beyond this initial linear elastic region, P‐5‐30 and P‐8‐30 experienced a soft‐elastic plateau (12.5% < ɛ < 50%), followed by a secondary linear elastic and strain‐hardening region (ɛ > 50%). Stress‐strain tests indicate that LC‐LCEs are stiffer than pristine LCEs below an inflection point. Beyond ɛ ≈ 125%, the elastic modulus of P0 surpasses that of P‐5‐30 and P‐8‐30. The elastic modulus (calculated at 185% < ɛ <  235%), elongation at break, and toughness at break for P‐5‐30 are calculated at ≈220 kPa, ≈300%, and 758 kJ m^−3^, while those of P‐8‐30 are calculated at ≈230 kPa, ≈330%, and 1198 kJ m^−3^. We argue that the larger content of LMWLCs and smaller content of elastic network in P‐5‐30 and P‐8‐30 result in P0 being overall stiffer, extensible, and tougher at very high strains.

Figure 4b shows the stress‐strain profile of monodomain samples, M0, M‐5‐30, and M‐8‐30. Elastic moduli of M‐5‐30 (≈13.4 MPa) and M‐8‐30 (≈18.3 MPa), calculated within their initial linear elastic region (ɛ < 20%) are significantly larger than that of M0 (≈2.1 MPa). While M0 roughly demonstrates monotonous stress‐strain behavior up to the failure, the linear elastic region in M‐5‐30 and M‐8‐30 comparably experiences a drastic slope change and softening before their failure. Notably, the strain at failure for M‐5‐30 and M‐8‐30 (≈40%) is much smaller than that of M0 (≈135%). The toughness of M‐5‐30 and M‐8‐30 at their failure (i.e., ≈40%) was calculated as 788 and 879 kJ m^−3^, respectively, which are larger than that of M0 calculated at 40% strain (212 kJ m^−3^). Though, when considering the overall toughness, M0 (1929 kJ m^−3^) outperforms LC‐LCEs on account of their reduced extensibility and elastic content.

Theoretically, the larger stiffness and toughness of monodomain LC‐LCEs should translate to their better ability to provide work and actuation stress. In line with this prediction, superior active thermal strokes (%) were measured for M‐5‐30 (28.5 ± 6.4) and M‐8‐30 (18.5 ± 0.7) compared to that of M0 (14.5 ± 3.5). Similarly, isometric thermal stress of M‐5‐30 (648 ± 28 kPa) and M‐8‐30 (726 ± 16 kPa) were greater than that of M0 (380 ± 14 kPa). (See details in Materials and Methods as well as Note S8, Supporting Information).

Our results strongly suggest that the inclusion of LMWLCs to LCEs significantly alters their strain‐stress behavior and dramatically stiffens them at moderate strains (ɛ < 40%). We attribute such stiffening to two mechanisms: phase separation and the emergence of induced smectic signatures. We postulate that the former has a more significant role in the stiffening of polydomain samples than in their monodomain counterparts. However, we are not certain about the extent to which each mechanism contributes to the overall stiffening. For polydomain samples, our results may be interpreted in the framework of extended Eshelby's theory, developed by Style and coworkers, where the inclusion of liquids in solids, counterintuitively, causes stiffening when the radius of liquid inclusions becomes smaller than the elastocapillary length. Considering *E*_*P*0_ < 30kPa, the elastocapillary length in P‐5‐30 systems can be estimated between 71 and 1000 nm (see Note S7, Supporting Information for details). TEM results show that the radius of phase‐separated domains is indeed smaller than the upper and comparable to the lower limits of estimated elastocapillary length, suggesting that the surface tension of LMWLC‐rich domains competes with and cloaks the far‐field stress applied to them. The presence of domains with smectic signature, as confirmed by DSC and DMA, also contributes to the stiffening observed in polydomain LC‐LCEs.

For monodomain samples, the elastic modulus significantly increases by photo‐polymerization and the second stage of cross‐linking. As a result, we expect the surface tension to have a less important role in stiffening, since the elastocapillary length of monodomain LCEs is expected to decrease drastically, perhaps even below the average radius of phase‐separated domains (see Note S7, Supporting Information for details). The presence of frozen‐in aligned smectic domains and local layered ordering, as confirmed by SAXS and WAXS, appears to have a more plausible contribution to the stiffening observed in monodomain LC‐LCEs. The stronger intermolecular interactions within the layers can act like physical crosslinks or reinforcing points, similar to semi‐crystalline polymers, which not only increase the stiffness but also limit the extensibility of the material.^[^ ^27^ ^]^ Stretching beyond the initial linear elastic region directs the stress to smectic phase‐separated domains, which leads to the dissociation of layers within these domains, with less stress.

## 3 Conclusion

In this work, we explored the impact of the inclusion of LMWLCs, namely 5CB and 8CB, into thiol‐acrylate LCEs and showed that the addition of LMWLCs, at a certain concentration, can dramatically alter the stress‐strain behavior of polydomain and monodomain host LCEs and significantly enhance their elastic modulus. We believe such macroscopic alterations originated from two separate but related mechanisms. First, post‐polymerization phase‐separation after the first stage of cross‐linking (thiol‐Michael addition) resulted in the emergence of LMWLC‐rich domains with an average radius comparable to the elastocapillary length. DSC and DMA results also confirmed the presence of induced smectic mesophase in these samples. Consequently, the tensile stress‐strain of polydomain LC‐LCEs shows an initial distinct linear elastic region. For samples doped with 5CB and 8CB, we recorded up to 13‐ and 30‐fold increases in elastic modulus, respectively, within this initial linear elastic region. This observation deviates from the original Eshelby's theory and aligns with the extended model developed by Style and coworkers, where the inclusion of liquid into solids cloaks the far‐field stress and impedes deformation if the radius of liquid droplets is smaller than the elastocapillary length.

Second, the far‐field strain applied to LC‐LCE samples not only leads to the alignment of LCE mesogens within the solid matrix but also the uniaxial ordering of clusters with smectic signature, promoting dipole‐dipole interaction between LMWLCs and the enhancement of short‐range smectic (cybotactic) domains. By the second stage of cross‐linking with light, monodomain LC‐LCEs benefit from the frozen‐in cybotactic ordering, which can act as additional physical cross‐linking points. Consequently, the tensile stress‐strain of monodomain LC‐LCEs shows an initial distinct linear elastic region followed by a softening. For samples doped with 5CB and 8CB, we recorded up to 6.5‐ and 9‐fold increases in elastic modulus, respectively, within this linear elastic region.

It is important to compare our system with other works employing alternative stiffening methods. The crystallized monodomain thiol‐ene nematic LCE developed by Ware and coworkers boasts substantially larger mechanical properties due to the prevalence of solid domains.^[^ ^7^ ^]^ Another strategy involving an interpenetrating polymer network (IPN) of separate acrylate and urethane LC networks was harnessed by Yang and coworkers.^[^ ^8^ ^]^ The reported elastic modulus of this LCE is smaller than ours (10.4 MPa), though this was measured above the LCE's clearing point. Aside from these reports to the best of our knowledge, our monodomain samples exhibit the highest stiffness of any reported LCE with liquid inclusion. Compared to prior results on the inclusion of liquid metals (EGaIn) in thiol‐acrylate LCEs, our monodomain LC‐LCEs demonstrate much larger and greater improvement of elastic modulus within their linear elastic region (ɛ < 20%). The maximum elastic modulus Ford and coworkers obtained from their polydomain LM‐LCE samples was ≈4.3 MPa.^[^ ^12^ ^]^ The maximum elastic modulus Lu and coworkers obtained from their monodomain LM‐LCE samples was ≈2.0 MPa.^[^ ^14^ ^]^ This is interesting since EGaIn has a much larger surface tension than 5CB and 8CB at room temperature and, intuitively, should stiffen the host LCE network more pronouncedly. It is also noteworthy that the elastic modulus of our system is desirable for artificial muscles,^[^ ^8^ ^]^ and unlike the reports mentioned above, the inclusion of LMWLCs in LCEs did not adversely affect their thermal strain or liquid crystalline order. In fact, the enhanced stiffness and molecular order of LC‐LCEs resulted in superior active thermal stroke, with work densities more than twice that of the control.

While offering the abovementioned advantages, there are shortcomings and open questions that can be the subject of an interesting future study. For instance, our LC‐LCEs show less overall extensibility and toughness compared to pristine LCEs here and the systems cited above. Moreover, the current study does not characterize in detail the kinetics of polymerization, and the effect of time on the phase‐separation, mechanical properties of LC‐LCEs, as well as the size and shape of phase‐separated domains. The time‐dependent nature of thiol‐Michael addition and LMWLC phase separation, which occur in the synthesis of our thiol‐acrylate LC‐LCEs, inherently bears batch‐to‐batch variability. A systematic investigation on these questions could hand in more robust synthetic pathways toward high‐performance LC‐LCEs. All in all, the inclusion of low molecular weight liquid crystals into LCEs provides a straightforward yet powerful approach to improving their mechanical properties, without sacrificing their liquid crystalline nature, i.e. order and thermal strain. We anticipate this facile approach to be applied in improving the mechanical properties of a variety of polymers to mitigate the conventional tradeoff between stiffness and extensibility.

## 4 Experimental Section

### Materials

1,4‐Bis[4‐(3‐acryloyloxypropyloxy) benzoyloxy]‐2‐methylbenzene (RM257) was purchased from Synthon Chemicals. 4‐Cyano‐4'‐pentylbiphenyl (5CB), 4‐Cyano‐4'‐octylbiphenyl (8CB), and 2,2‐Dimethoxy‐2‐phenylacetophenone (IG 651) were purchased from TCI Chemicals. Trichloro(1H,1H,2H,2H‐perfluorooctyl)silane, 2,2 ‐(Ethylenedioxy)diethanethiol (EDDET), Pentaerythritol tetrakis(3‐mercaptopropionate) (PETMP), 4‐methoxyphenol (MEHQ), dipropylamine (DPA), and chloroform were purchased from Sigma–Aldrich. All chemicals were used as received.

### Cell Preparation

Glass slides were cleaned with acetone, isopropanol, and deionized water followed by 15 min of UV ozone cleaning. Hydrophobic glass slides were produced from silanization by evaporating 1–2 droplets of trichloro(1H,1H,2H,2H‐perfluorooctyl)silane under vacuum for 90 min.

### LCE Synthesis

The polydomain base formulation (P0) was prepared with modification from a previously published method as follows.^[^ ^18^ ^]^ A precursor solution was prepared of PETMP (28.3 mg), EDDET (63.3 mg), RM257 (300 mg), MEHQ (0.8 mg), and chloroform (120 mg). MEHQ acts as an inhibitor to prevent vinyl‐vinyl reaction prior to UV curing. This was heated until all components were dissolved, and then the photoinitiator IRG 651 (1.5 mg) was added. To initiate the base‐catalyzed thiol‐Michael addition, a 2 wt.% catalyst solution of DPA in chloroform (19 µL total) was added to the precursor mixture. This was followed by immediate vortexing and placement under a vacuum for bubble removal. The mixture was promptly filled into 0.5 mm thick cells on a hot plate at 64 °C via capillary filling. Then the cell was removed from the hot plate and the reaction proceeded for three days at room temperature (23 °C). Finally, the top glass slide was removed with a razor, and the sample was placed under vacuum (25 inHg) for 12 h to remove chloroform and quench the reaction. Polydomain LC‐LCEs (P‐5‐30 and P‐8‐30) followed the same synthesis as the control (P0), except 118.2 mg of either 5CB or 8CB were added to the precursor mixture before dissolving, followed by 30 µL of catalyst solution instead of 19 µL (this was necessary for the reaction to complete within three days). Monodomain LCEs were made through uniaxially straining polydomain samples in a tensile machine (Cellscale Univert) to the desired length and then photopolymerizing with one 365 nm UV light on either side at a distance of 6 cm (Spectroline E‐Series).

### Differential Scanning Calorimetry

Differential Scanning Calorimetry was conducted on a TA Instruments Q2000 with RCS90 on 7–9 mg samples. Samples underwent a heating and cooling cycle to reset the thermal history followed by a heating cycle from which measurements were taken. The ramp rate was 5 °C min^−1^.

### Dynamic Mechanical Analysis

Dynamic Mechanical Analysis (DMA) was performed on a TA Instruments Q800 following a previously published protocol.^[^ ^11^ ^]^ Briefly, 15 × 4 × 0.5 mm^3^ specimens were measured in tensile mode under 0.2% strain and 1 Hz with a heating rate of 3 °C min^−1^ from −80 °C to 120 °C. The force track was set to 125%.

### Thermal Expansion

Thermal Expansion (TE) measurements were taken on a custom‐made heating stage (Polaviz) with a Dino‐Lite Edge 3.0 USB microscope. 2 × 2 mm monodomain specimens for each formulation were heating at 3 °C min^−1^ from 25 °C to 140 °C and then cooled down to the starting temperature. Photos were taken every 5 °C, and edge length changes were measured in Tracker (Physlets.org).

### Small Angle X‐Ray Scattering

Small‐angle X‐ray Scattering (SAXS) was performed in the Advanced Materials and Liquid Crystal Institute of Kent State University on Xenocs Xeuss 3.0 SAXS/WAXS imaging beamline with an Eiger2 R 1M detector equipped with a Cu K_α_ source producing 8.05 *keV* X‐rays (1.542 Å wavelength). The beam size at the sample was 0.9 *mm*, the sample‐detector distance was 140 *mm*, and the detector's pixel size was 75*mm* × 75*mm*. The detector's active area (including detector gaps) was 77.1 *mm* × 79.65 *mm*. The q‐space range was measured from 0.0002 to 0.3 Å^−1^. The SAXS patterns were processed using Xenocs’ XSACT software to obtain the azimuthally averaged scattered intensity, *I(q)*. The datasets were fitted in OriginPro to determine d‐spacings and correlation lengths. Identical smoothing and baseline correction procedures were applied to all the datasets, without modifying peak heights and FWHM, for plotting purposes.

### Wide‐Angle X‐Ray Scattering

Wide‐Angle X‐ray Scattering (WAXS) was performed in the Advanced Materials and Liquid Crystal Institute of Kent State University on a Xenocs Xeuss 3.0 SAXS/WAXS imaging beamline with an Eiger2 R 1M detector equipped with a Cu K_α _source producing 8.05 keV X‐rays (1.542 Å wavelength). The sample‐detector distance was 42.5 mm, the incident angle 0.099975°, and q‐space range: 0.003–3.89 Å^−1^_._ The WAXS patterns were processed using Xenocs’ XSACT software to obtain the azimuthally averaged scattered intensity, *I(q)*. The datasets were fitted in OriginPro to determine d‐spacings and correlation lengths. Identical smoothing and baseline correction procedures were applied to all the datasets, without modifying peak heights and FWHM, for plotting purposes.

### Transmission Electron Microscopy (TEM)

M‐5‐30 specimens were embedded in‐plane in Spurr's epoxy resin overnight at 50 °C. Thin sections (70 nm) were cut on a Leica UCT ultramicrotome and picked up onto Cu grids. Some of the grids were exposed to either Osmium tetroxide vapors or Ruthenium tetroxide vapors for ≈1.5 h. The grids were viewed in a JEOL JEM 1200 EX TEMSCAN transmission electron microscope (JEOL, Peabody, MA, USA) operating at an accelerating voltage of 80 kV. Images were acquired with an AMT 4‐megapixel digital camera (Advanced Microscopy Techniques, Woburn, MA). Some of the samples were also viewed with a Thermo Fisher Scientific Talos 200X operated at 200 kV.

### Tensile Tests

For polydomain LCEs, 12 × 4 × 0.5 mm^3^ (length × width × thickness) specimens with gauge length 6 mm were strained in a Cellscale Univert equipped with a 10 N load cell at 0.1 mm·s^−1^ until failure with 0.1 N preload. Monodomain tensile tests in the direction parallel to alignment were performed with the same strain rate as polydomain samples but with different lengths (16 mm), widths (4 mm), gauge lengths (5 mm), and load cell (50 N). Thicknesses for formulations depended on the strain at which they were photopolymerized; these were measured before tensile testing with a caliper. To prevent slippage in the monodomain samples, 60‐grit sandpaper was fastened to the flat of the tensile clamp jaws with double‐sided tape. Note that the elastic modulus was measured at 185% < ɛ <  235% as all samples showed a full transition from poly to monodomain. All SAXS and WAXS data were also collected for samples that experienced such strains. It was also important to note that for all P‐5‐30 and P‐8‐30 samples, the linear elastic region appears 48 h after the first stage of polymerization, and peaks 5 days after the polymerization. P0 does not show a tangible linear elastic behavior with time, but becomes stiffer in general. This confirms that the phase separation and the size of domains increase over time.

### Active Thermal Stroke

The testing apparatus is shown in Figure S11 (Supporting Information). Monodomain specimens were cut to ≈12 × 5 mm pieces, and a gauge length of 5 mm was marked along the length of the specimen, parallel to the mesogen alignment, with two dots. The samples were weighed to find their mass, m. Before testing, a sample was hung from the top grip and its photo was taken, then a 75 g weight was attached to the bottom of the sample, and another photo was taken. The two photos were used to determine the displacement of the two dots after loading. During testing, the apparatus was heated while video orthogonal to the strain direction was taken. The air temperature around the sample was recorded every 10 s with a thermocouple until it reads 90 °C. Post‐testing, the displacement of the two dots vs time was measured using a video tracking software and can be approximately related to temperature using the temperature vs time correlation. Work density (J kg^−1^) was calculated by the equation $W_{d}=\frac{F_{g}d}{m}\;$, where F_g_ is the force applied by the weight (in Newtons) due to gravity, d is the total displacement of the weight (in meters), and m is the mass of the specimen (in kilograms).

### Isometric Thermal Stress

Monodomain specimens were cut to ≈12 × 4 mm pieces and a gauge length of 5 mm was marked along the length of the specimen, parallel to the mesogen alignment, with two dots using a marker. Their thicknesses were measured using calipers. Specimens were loaded onto a Cellscale Univert equipped with a 10 N load cell, and the gripper distance was manually adjusted to the point just before any stress would be applied to the material. The specimen would then be heated with a heat gun positioned at a fixed distance away until the force read by the load cell achieved a steady state. The isometric force resulting from heating was divided by the initial cross‐sectional area of the specimen to calculate the isometric thermal stress.

## Conflict of Interest

The authors declare no conflict of interest.

## Supporting information



Supporting Information

## Data Availability

The data that support the findings of this study are available in the supplementary material of this article.
