# Supplementary material for: Stiffening Liquid Crystal Elastomers with Liquid Crystal Inclusions
Source: Adv Mater. 2025 Jun 9;37(45):2504592. doi: 10.1002/adma.202504592 (PMC12617047; doi:10.1002/adma.202504592)
Supplement: Supplementary file 1 — Supporting Information [file ADMA-37-2504592-s001.docx]

Supplementary Information

Stiffening Liquid Crystal Elastomers with Liquid Crystal Inclusions

Sahad Vasanji^#^, Matthew Gene Scarfo^#^, Arwa Alyami, Tizazu Mekonnen, Parsin Hajireza, Mohand O. Saed, Antal Jákli, and Hamed Shahsavan*.

S. Vasanji, M. G. Scarfo, Prof T. Mekonnen., Prof H. Shahsavan.

Department of Chemical Engineering

Institute for Polymer Research

Center for Bioengineering and Biotechnology

Waterloo Institute for Nanotechnology

P. Hajireza

Department of Systems Design Engineering

Center for Bioengineering and Biotechnology

University of Waterloo, Waterloo, ON N2L 3G1, Canada
# Authors with equal contribution

* Corresponding author: E-mail: [hshahsav@uwaterloo.ca](mailto:hshahsav@uwaterloo.ca)

Dr. M. O. Saed.

Cavendish Laboratory

University of Cambridge

Cambridge, CB3 0HE, UK

Dr. Arwa Alyami, Prof. A. Jakli.
Department of Physics and Advanced Materials and Liquid Crystal Institute, Kent State University, Kent, Ohio 44242, USA

Table of Contents:

Supplementary Note 1: Characterization of 5CB Miscibility with LCE Matrix

Supplementary Figure 1: Characterization of 5CB Miscibility with LCE Matrix

Supplementary Table 1: Solubility Parameters and Differences for 5CB and LCE

Supplementary Figure 2: DSC Cooling Cycles for 5CB Formulations.

Supplementary Table 2: Transition Temperatures from DSC Cooling Cycles

Supplementary Figure 3: DMA Temperature Curves for 5CB Formulations

Supplementary Note 2: Poly- to Monodomain Transition Strain

Supplementary Figure 4: Schematic (left) and Setup (right) of the Transmission Measurement

Supplementary Figure 5: 1D Integrated Intensities for LC-LCEs

Supplementary Note 3: Strain Dependence and Calculation of Hermann’s Order Parameter

Supplementary Table 3: Strain-Dependent Order Parameters

Supplementary Note 4: Smectic Ordering via Strain-Induced Charge Transfer

Supplementary Figure 6: FTIR Spectra Before and After Stretching

Supplementary Note 5: Data Processing and Fitting of SWAXS Peaks

Supplementary Table 4: d-spacings and Correlation Lengths of *p1*, *p2*, *p3*

Supplementary Figure 7: Peak Fitting of SAXS

Supplementary Figure 8: Deconvolution and Peak Fitting of M-5-30 WAXS

Supplementary Figure 9: Deconvolution and Peak Fitting of M-8-30 WAXS

Supplementary Note 6: Investigation of LC-LCE Time-Dependent Mechanical Properties

Supplementary Figure 10: Time-Dependency of LER observed in LC-LCEs

Supplementary Note 7: Estimating the Elastocapillary Length

Supplementary Note 8: Estimating the Isotonic Work Density and Isometric Thermal Stress

Supplementary Table 5: Summary of Active Thermal Stroke Testing Data

Supplementary Figure 11: Performing the Active Thermal Stroke

Supplementary References

**Supplementary Note 1: Characterization of 5CB Miscibility with LCE Matrix**

Stiffening of solids with liquid inclusion was tested on solids doped with immiscible liquids with their host matrix ^[1]^. To examine 5CB’s miscibility with the LCE, swelling behavior (Figure S1a), dopant leakage (Figure S1b), contact angle measurements (Figure S1c), and group contribution theory (Figure S1d) are conducted as qualitative and quantitative metrics of solvent-polymer interactions.


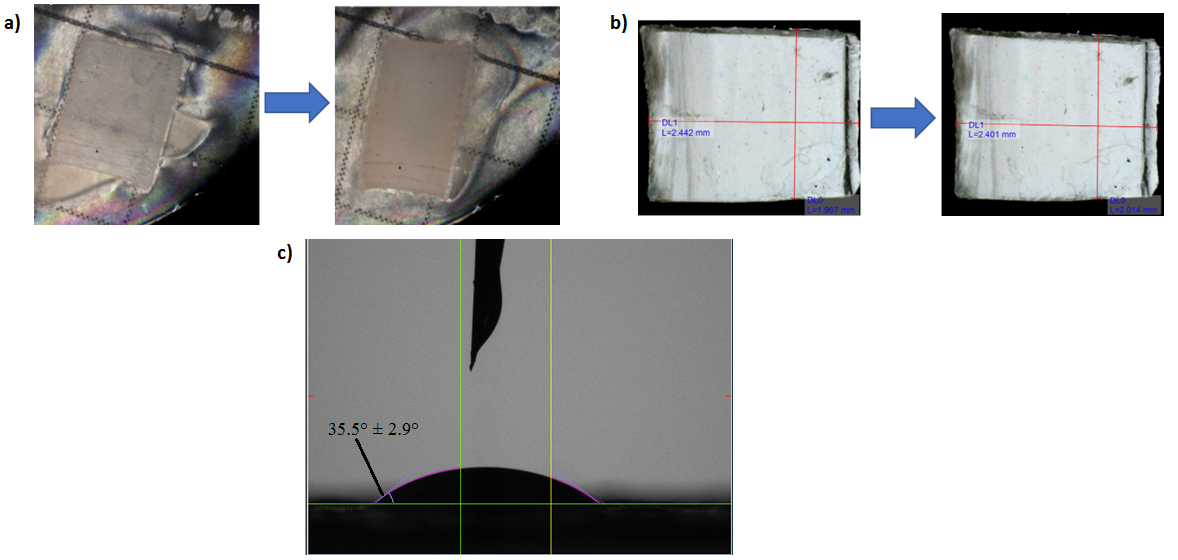


**Figure S1. Characterization of 5CB Miscibility with LCE matrix.** a) Swelling of M0 with 5CB by 135% after 20 hours. b) Minor length changes of M0 after 33 hours infer minimum dopant leakage. c) Contact angle of 5CB with P0 of 35.5˚ indicating good wettability.

The degree to which 5CB can swell the LCE qualitatively describes the dopant miscibility with its host matrix. This is especially important to inform the liquid inclusion stiffening hypothesis because 5CB substantially swells acrylate-based LCEs, as evidenced in the literature by swelling ratios of 110-246% ^[2–4]^. M0 was selected for testing since the isotropic swelling of polydomain LCE would be less observable ^[5]^. Positive (16%) and negative (17%) length changes were measured in the direction perpendicular and parallel to the director **n**, respectively (Figure S1a). Assuming the same length change in the direction perpendicular to alignment occurs along the sample thickness, the calculated swelling degree is 135%, which is in the lower half of 5CB’s swelling range of acrylate-based LCEs. Notably, the specimen observes conservation of volume, and minor discrepancies between this phenomenon and the measured percentages are attributed to the LCE edges not being exactly normal to the microscope. The graph paper was visible through the 5CB, as it exists in the nematic phase at room temperature.

The strength of cyanobiphenyl interactions with the LCE matrix was qualitatively estimated by measuring sample length changes due to dopant leakage over 33 hours. Sample M-5-70 was tested only, assuming that the lower 5CB loaded samples would have similar or less leakage. Minor positive (2%) and negative (2%) length changes are observed parallel and perpendicular to the alignment, suggesting minimal leakage of 5CB (Figure S1b).

Contact angle measurements provide an indication of miscibility, wherein the solvent wetting of the neat LCE confirms thermodynamically favorable interactions. The left and right contact angles of ten 5CB droplets on sample P0 were averaged to obtain 35.5° ± 2.9°, indicating good wettability and inferring good miscibility of 5CB with the LCE (Figure S1c).

Table S1: Solubility Parameters and Differences for 5CB and the LCE

| Component | $\boldsymbol{\delta}_{\boldsymbol{p}}{\boldsymbol{(}\boldsymbol{cal\cdot}\boldsymbol{cm}^{\boldsymbol{-3}}\mathbf{)}}^{\boldsymbol{0.5}}$ | $\boldsymbol{\delta}_{\boldsymbol{s}} {\boldsymbol{(}\boldsymbol{cal\cdot}\boldsymbol{cm}^{\boldsymbol{-3}}\mathbf{)}}^{\boldsymbol{0.5}}$ | ${\mathbf{(}\boldsymbol{\delta}_{\boldsymbol{s}}\boldsymbol{-}\boldsymbol{\delta}_{\boldsymbol{p}}\boldsymbol{)}}^{\boldsymbol{2}}$ |
| --- | --- | --- | --- |
| **LCE** | 9.52 | - | - |
| **5CB** | - | 10.01 | .24 |
| **Glycerol** | - | 16.5 | 48.72 |

Group Contribution Theory (GCT), developed by Small, provides a framework for determining solvent miscibility with a polymer using Molar Attraction Constants ^[6]^. As seen in Table S1, the difference between 5CB and the LCE solubility parameters is markedly less than the cutoff criterion of 1.2 cal·cm^3^ for the LCE to be soluble in the solvent. This reinforces the miscibility between the dopant and the matrix determined. As a counterexample, a solvent such as glycerol is expected to be immiscible since its $\delta_{s} {=16.5 (cal\cdot{cm}^{-3})}^{0.5}$, substantially differs from that of the neat LCE and exceeds the solubility criterion. Note that no Molar Attraction Constants were found for a trisubstituted phenyl, as is the case with the central benzene in RM257. Instead, the Molar Attraction Constant for phenylene was used to approximate this instance, with the methyl group also added. Considering the Molar Attraction Constants tabulated in Small’s work, a trisubstituted phenyl ring would not result in failing the cutoff criterion.


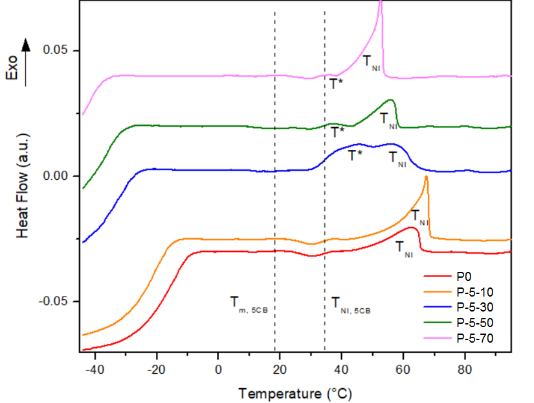

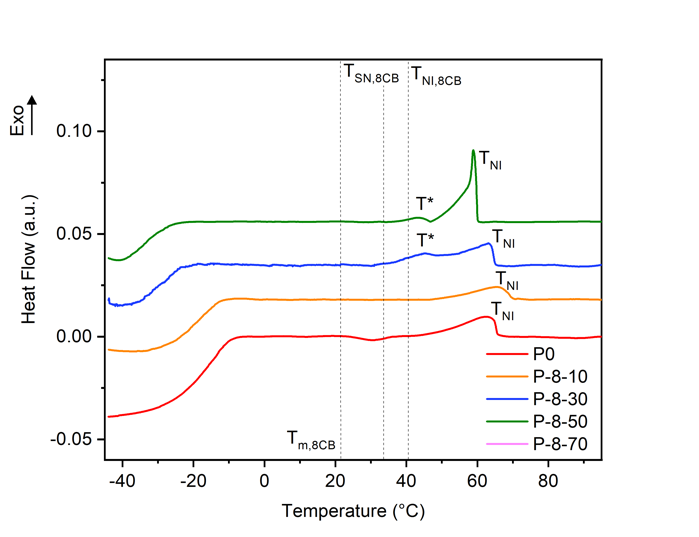


**(d)**

**(b)**

**(a)**


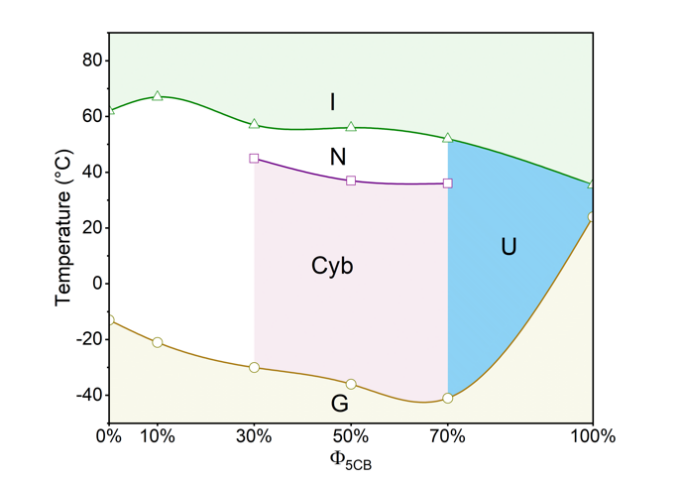

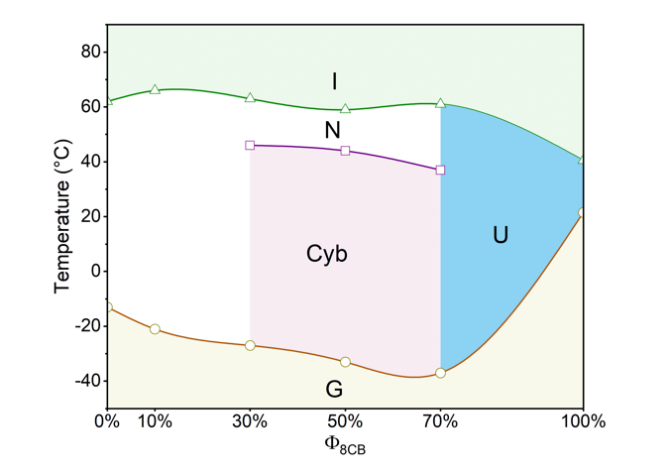


**(c)**

**Figure S2:** **DSC Cooling Cycles for 5CB and 8CB Formulations**. Endothermic transitions are labeled below their peaks. Pure 5CB’s and 8CB’s transitions are denoted with dashed lines in Figure S2 (a) and (b), respectively. DSC of P-8-70 turned out to be noisy and is not shown here. That said, we could still extract the transition temperatures. Phase diagrams of LC-LCEs constructed based on the DSC (cooling cycle) of (c) P-5-Z and (d) P-8-Z samples. Different regions show the mesophase elastomers experience at different temperatures: above their T_NI_ (i.e., isotropic or I) is shown by green, between their T_NI_ and T^*^ (i.e., nematic or N) is shown by white, between T^*^ and Tg (i.e., cybotactic or Cyb) is shown by purple, and below their Tg (i.e., glassy or G) is shown by brown. Regions where we do not have confidence in the type of mesophase (i.e., unknown or U) are shaded in blue.

Table S2: Transition Temperatures from DSC Cooling Cycles

*T_cc_ = cold crystallization transition, T* = additional peak due to high 5CB/ 8CB loading, T_NI_ = nematic-isotropic transition*

|  | T_CC_ (°C) | T* (°C) | T_NI_ (°C) |
| --- | --- | --- | --- |
| P0 | 30 | - | 62 |
| P-5-10 | 30 | - | 67 |
| P-8-10 | - | - | 66 |
| P-5-30 | 30 | 45 | 57 |
| P-8-30 | - | 46 | 63 |
| P-5-50 | 30 | 37 | 56 |
| P-8-50 | - | 44 | 59 |
| P-5-70 | 30 | 36 | 52 |
| P-8-70 | - | 37 | 61 |

**
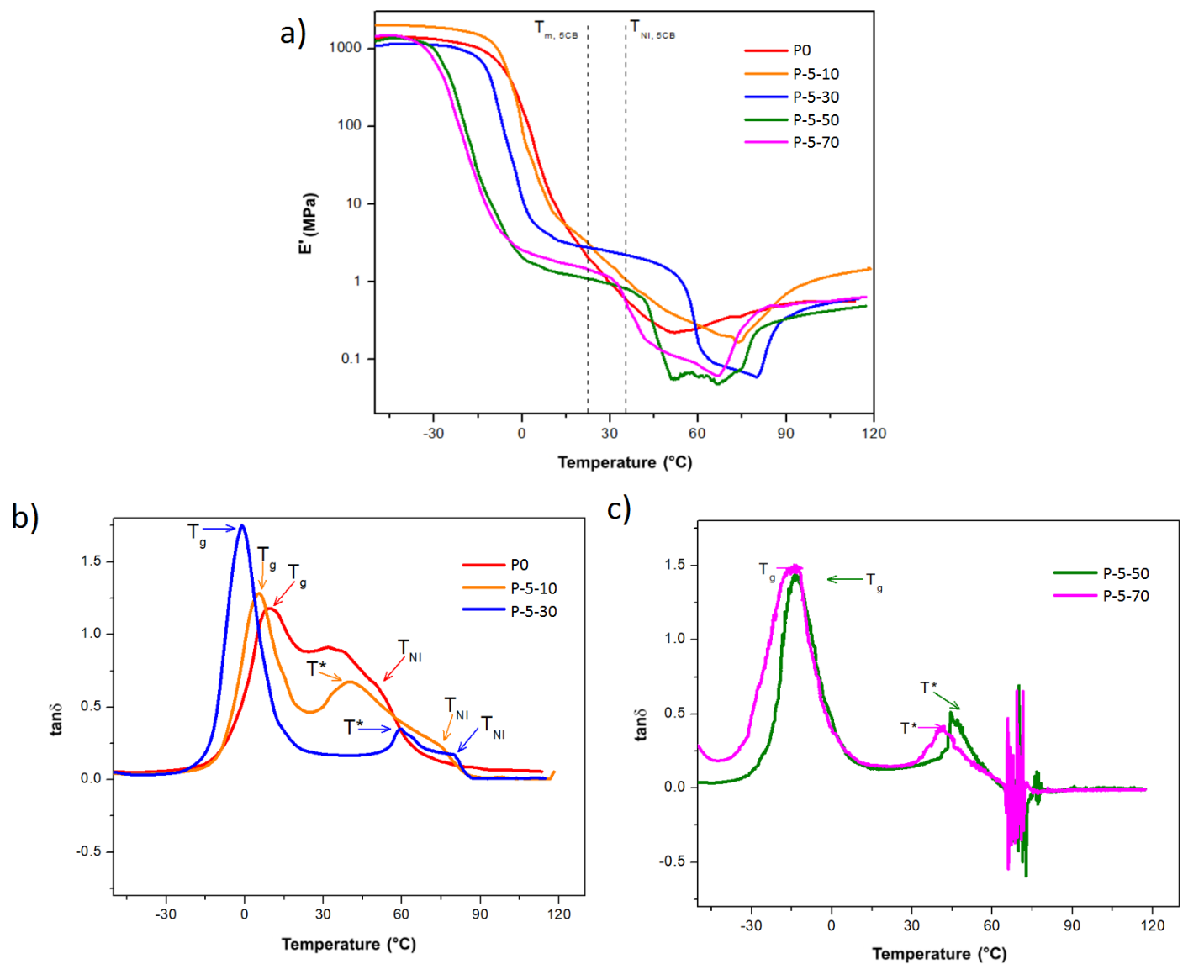
**

**Figure S3. DMA Temperature Curves for 5CB Formulations.** Glass transition temperatures (T_g_), additional peaks (T*), and clearing points (T_NI_) are labeled on each graph. a) Storage moduli (E’) as a function of temperature. b), c) tan(δ) as a function of temperature. Note that T_NI_ for P-5-50 and P-5-70 could not be measured due to substantial noise during measurement.

**Supplementary Note 2: Poly- to Monodomain Transition Strain**

The polydomain to monodomain transition (PMT) was quantitatively measured by tracking changes in the transmission of linearly polarized light (T) with respect to strain (%). Since mesogens are anisotropic, the polarized light’s transmittance should change as they reorient under strain. The Transmission Coefficients were measured with small modifications to a previously reported protocol ^[7]^. The transmission coefficient is calculated as $T=\frac{P}{P_{0}}$, where $P$ is the power of the transmitted light, and $P_{0}$ is the incident power of the light source. A custom setup shown in Figure S4 was used, which is comprised of a linearly polarized continuous wave laser (637nm), alignment mirrors, a wave plate for rotating the laser's polarization, a telescoping lens to focus the laser on the sample's center, the LCE loaded into the tensile machine, and an optical power meter. An operating power of 1 mW was used, and the unobstructed beam’s power was measured and averaged for 1 minute to obtain the incident power. An LCE specimen was then loaded into the tensile machine (gauge length 6mm) such that the beam was centered on the LCE to block the light’s path to the detector. The sample was preloaded with 0.1 N and then strained at 0.1 mm.s^-1^ until failure, while simultaneously measuring the power incident on the detector every 0.5 seconds. The transmission coefficient for light polarization parallel and perpendicular to the strain direction was calculated as the ratio of transmitted power to the incident power and is shown in Figure S4.


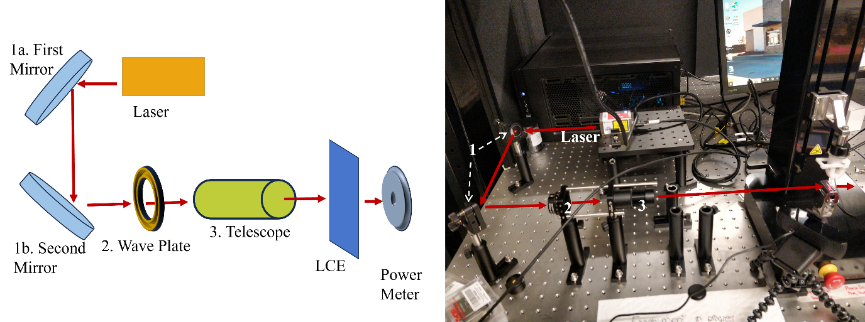

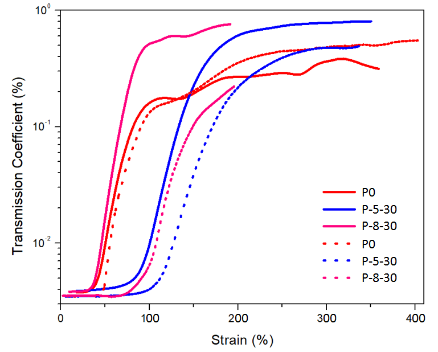


(b)

(a)

**Figure S4.** (a) Schematic (left) and setup (right) of the transmission measurement. The power meter is to the right of the tensile apparatus (not pictured). (b) Strain Dependence of Transmission Coefficient. Solid (dashed) lines show the parallel (perpendicular) coefficients.

Sharp initial slopes in the transmission indicate the initial ordering of mesogens, at strains that approximately correspond to a soft elastic plateau. This data supports the qualitative examination during WAXS studies of the strain required for inducing the PMT. It also provides strains beyond which the formulations should be stretched & programmed to ensure a fully monodomain LCE is obtained. 235% was selected as the programming strain for P-5-30, while 194% was decided upon for P0. This decision was made because beyond that strain the parallel transmission coefficient roughly begins to plateau, just as with 235% in P-5-30.

Compared to P-5-30, P-8-30 displays even greater anisotropy in the onset of light transmission through the LCE. We suspect this phenomenon is due to 8CB-rich domains within the LCE existing as larger regions than the 5CB domains in P-5-30. P-5-30 and P-8-30’s transmission anisotropy is contrasted by the control LCE, which is seen to have similar transmission onsets in parallel and perpendicular directions. The perpendicular transmission coefficient for P-8-30 was not observed to reach a plateau. Since the opaque-transparent transition’s onset is most associated with the onset strain for the parallel transmission coefficient, P-8-30 being programmed beyond 96% strain would elicit a fully monodomain LCE. One might notice that the failure strain for P-8-30 in this dataset is smaller than in the polydomain tensile data (Figure S10)—this is due to the inconsistent nature of how LC domains reorient. Since polydomain formation is a stochastic process, different batches of LCE will be polydomain to different extents, resulting in different degrees of domain rotation and extensibility. Considering this, 185% was selected as the programming strain for P-8-30 (i.e., where the parallel transmission coefficient begins its final plateau).

**
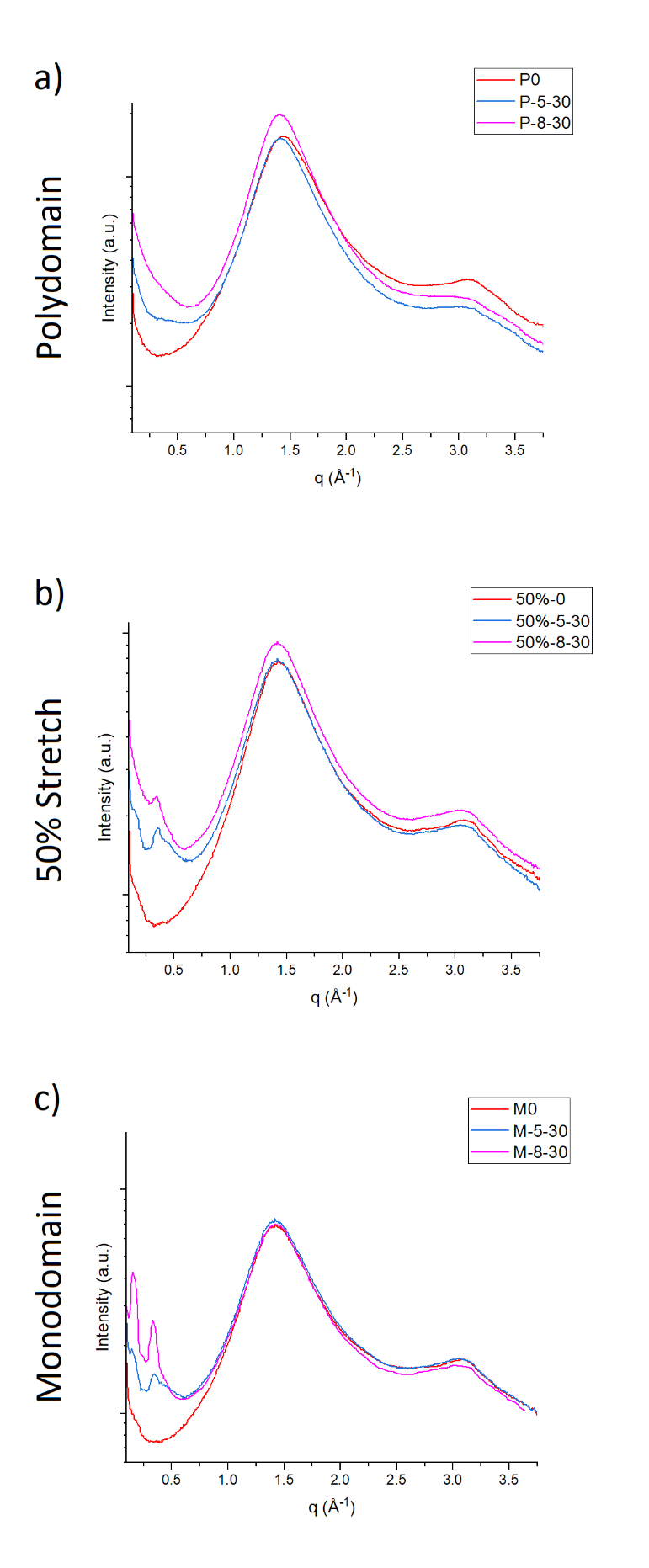
**

**Figure S5. 1D Integrated Intensities for LC-LCEs.** Anisotropic diffraction peaks p1, p2, and p3 are labeled.

**Supplementary Note 3: Strain Dependence and Calculation of Hermann’s Order Parameter**

A quantitative explanation of the extent of mesogen alignment within each LCE was determined by the orientational order parameter, *S*, calculated from azimuthally integrated 1-dimensional (1D) curves of the 2D WAXS patterns using Herman’s orientation function ^[8]^:

$$\begin{aligned} \boldsymbol{S=}\frac{\boldsymbol{3}\left\langle\mathbf{cos}^{\boldsymbol{2}} \boldsymbol{\theta} \right\rangle\boldsymbol{-1}}{\boldsymbol{2}} \end{aligned}$$

Where *θ* is the azimuthal angle. The expectation value in the above equation is calculated from the azimuthal angle and corresponding intensities *I(ø)*:

$$\begin{aligned} \left\langle\boldsymbol{cos\theta} \right\rangle\boldsymbol{=}\frac{\int_{\boldsymbol{0}}^{\frac{\boldsymbol{\pi}}{\boldsymbol{2}}} \boldsymbol{I}\left( \boldsymbol{\emptyset} \right)\boldsymbol{sin\emptyset}\mathbf{cos}^{\boldsymbol{2}} \boldsymbol{\emptyset d\emptyset}}{\int_{\boldsymbol{0}}^{\frac{\boldsymbol{\pi}}{\boldsymbol{2}}} \boldsymbol{I}\left( \boldsymbol{\emptyset} \right)\boldsymbol{sin\emptyset d\emptyset}} \end{aligned}$$

Values in Table S3 are tabulated in the same arrangement as diffraction patterns in Figure S5, apart from P-8-30 tabulated in the last row. Strain 1 and Strain 2 correspond to no strain and 50% strain, respectively, while Strain 3 is the minimum requisite strain for becoming visibly transparent (i.e., monodomain).

**Table S3: Strain-Dependent Order Parameters**

| Formulation (Strain) | | Strain 1 | Strain 2 | Strain 3 |
| --- | --- | --- | --- | --- |
| P0 | (0%, 50%, 60%) | 0.34 | 0.64 | 0.63 |
| P-5-30 | (0%, 50%, 235%) | 0.28 | 0.65 | 0.67 |
| P-8-30 | (0%, 50%, 185%) | 0.14 | 0.62 | 0.66 |

Interestingly, P-5-30 and P-8-30 initially have small *S* values but evolve to have the largest order parameters compared to P0. P-8-30 displays the smallest value for *S* in the polydomain state of all tested formulations. As smectic regions not aligned with the director detract from the order parameter more than unaligned nematic regions, P-8-30 sensibly yields a smaller polydomain order parameter. Through straining to 50%, P-8-30’s order parameter increases to a lesser extent than the control or P-5-30, a trend likely due to the higher fraction of smectic domains having a larger entropy barrier to overcome for rotation and realignment ^[9]^. Only at high strains does P-8-30’s order parameter become the second largest after P-5-30, exceeding the values of all formulations. It makes sense that smectic domains aligned with the director reinforce the stretched LCE chains such that an augmented order parameter is observed. However, P-5-30’s *p­­_3_* is still narrower on account of 5CB’s nematicity. Overall, it is observed that a higher degree of smecticity can reinforce chain alignment and give rise to a larger order parameter.

**Supplementary Note 4: Smectic Ordering via Strain-Induced Charge Transfer**

Comparing the change in labeled wavenumbers for ν_CN_ across the polydomain-monodomain transition (PMT) this frequency decreases 1.63 cm­­_­_^-1^ and 3.61 cm^-1^ for P-5-30 and P-8-30, respectively. P-8-30’s cyano group absorbance frequency reduces more than double that of P-5-30, confirming larger charge redistribution in the 8CB loaded formulation. This is because 8CB is more prone to close packing than 5CB since it has larger van der Waals interactions from the longer alkyl chain. 8CB can more readily adopt the dimeric arrangement, producing stronger charge transfer and dipole-dipole/induced dipole interactions when compressed laterally from chain stretching. A shorter d-spacing is thus created, as observed with M-8-30’s d-spacing (~ the 8CB dimer, 37Å) and M-5-30’s d-spacing (~ two 5CB molecular lengths, 37 Å).


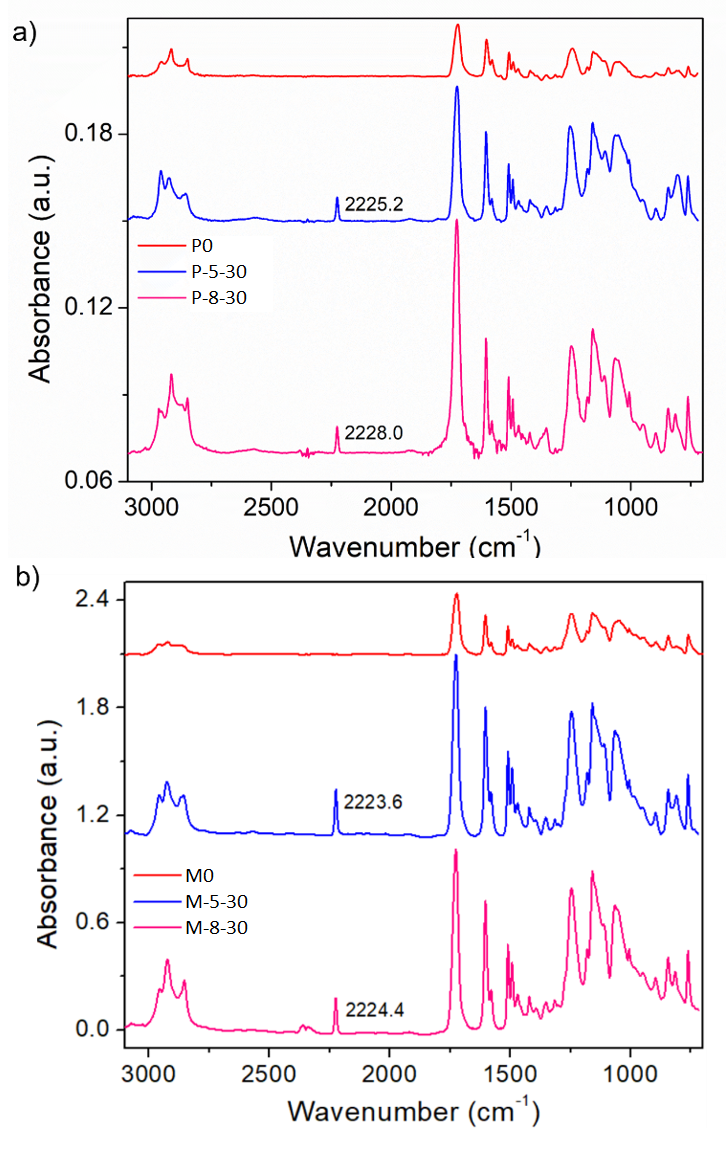


**Figure S6: FTIR Spectra Before and After Stretching.** a) Polydomain LCEs vs. b) monodomain LCEs. Cyano-group absorption frequencies are labeled.

**Supplementary Note 5: Data Processing and Fitting of SWAXS Peaks**

To determine d-spacings ($d_{i,x}$) and correlation lengths ($\xi_{i,x}$), 1D integrated intensities of SWAXS data were analyzed using the Multiple Peaks Fitting module in OriginPro. For ease of deconvolution, *p_1_* and *p_2_* of WAXS were fitted separately from *p_3_*.

Non-linear Gaussian fits were applied in OriginPro to *p1* of SAXS and *p1* and *p2* of WAXS. To achieve the most accurate fit of *p3*, a Pseudo-Voight function was used, deconvoluting two other peaks that are attributable to amorphous components of the system. The peak centers and FWHMs, denoted as xc and w in Figures S7-9, were used to calculate d-spacings and correlation lengths, respectively, as tabulated in Table S4.

**Table S4: d-spacings and Correlation Lengths for *p1*, *p2*, *p3***

|  | $\boldsymbol{d}_{\mathbf{3,}\boldsymbol{x}}\mathbf{(Å)}$ | $\boldsymbol{d}_{\mathbf{2,}\boldsymbol{x}}\mathbf{(Å)}$ | $\boldsymbol{d}_{\mathbf{1,}\boldsymbol{x}}\mathbf{(Å)}$ | $\boldsymbol{\xi}_{\mathbf{3,}\boldsymbol{x}} \mathbf{(Å)}$ | $\boldsymbol{\xi}_{\boldsymbol{1}\mathbf{,}\boldsymbol{x}}\boldsymbol{/SAXS}\mathbf{(Å)}$ | $\boldsymbol{\xi}_{\mathbf{1,}\boldsymbol{x}}\mathbf{/WAXS(Å)}$ |
| --- | --- | --- | --- | --- | --- | --- |
| **M0** | 4.5 | - | - | 13.3 | - | - |
| **M-5-30** | 4.5 | 18 | 37 | 13.5 | 2204 | 93 |
| **M-8-30** | 4.5 | 18 | 37 | 13.5 | 2087 | 122 |

**
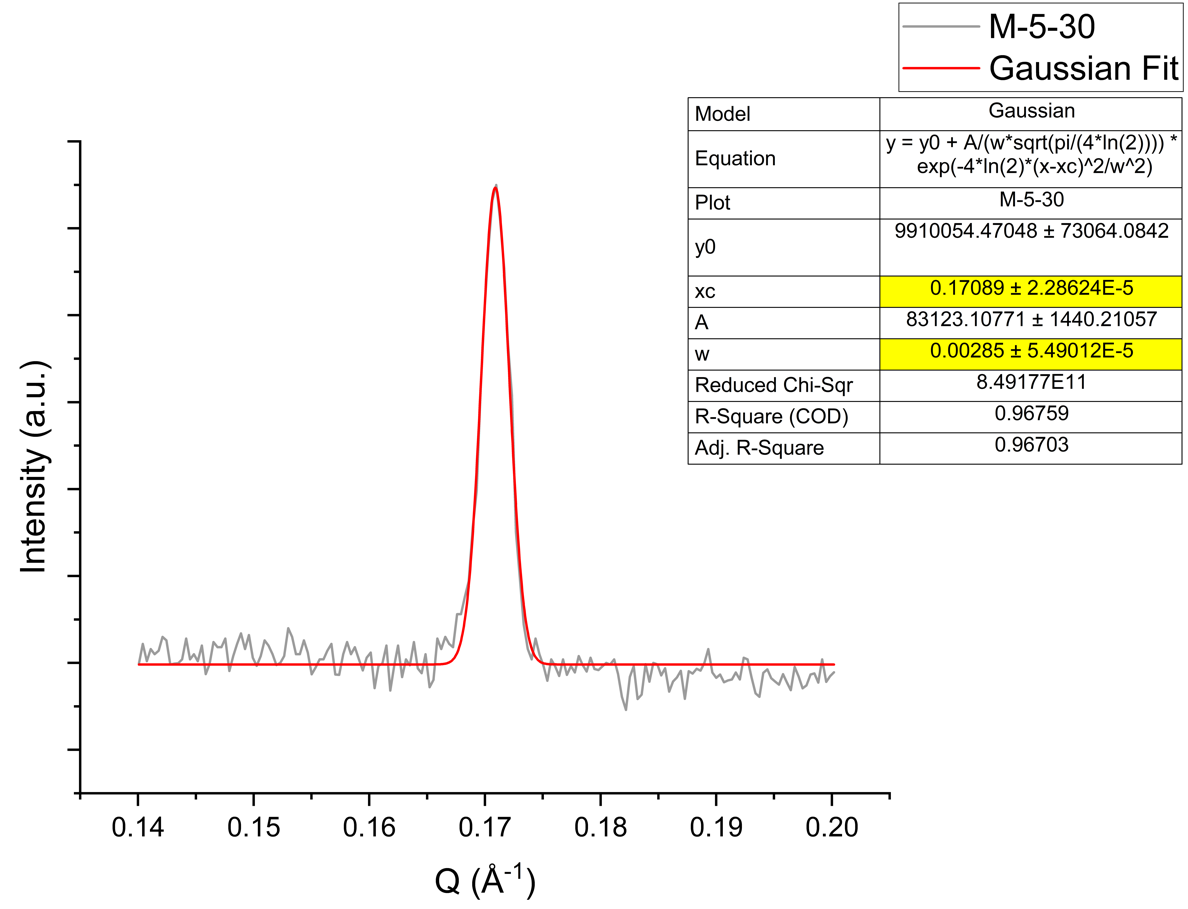

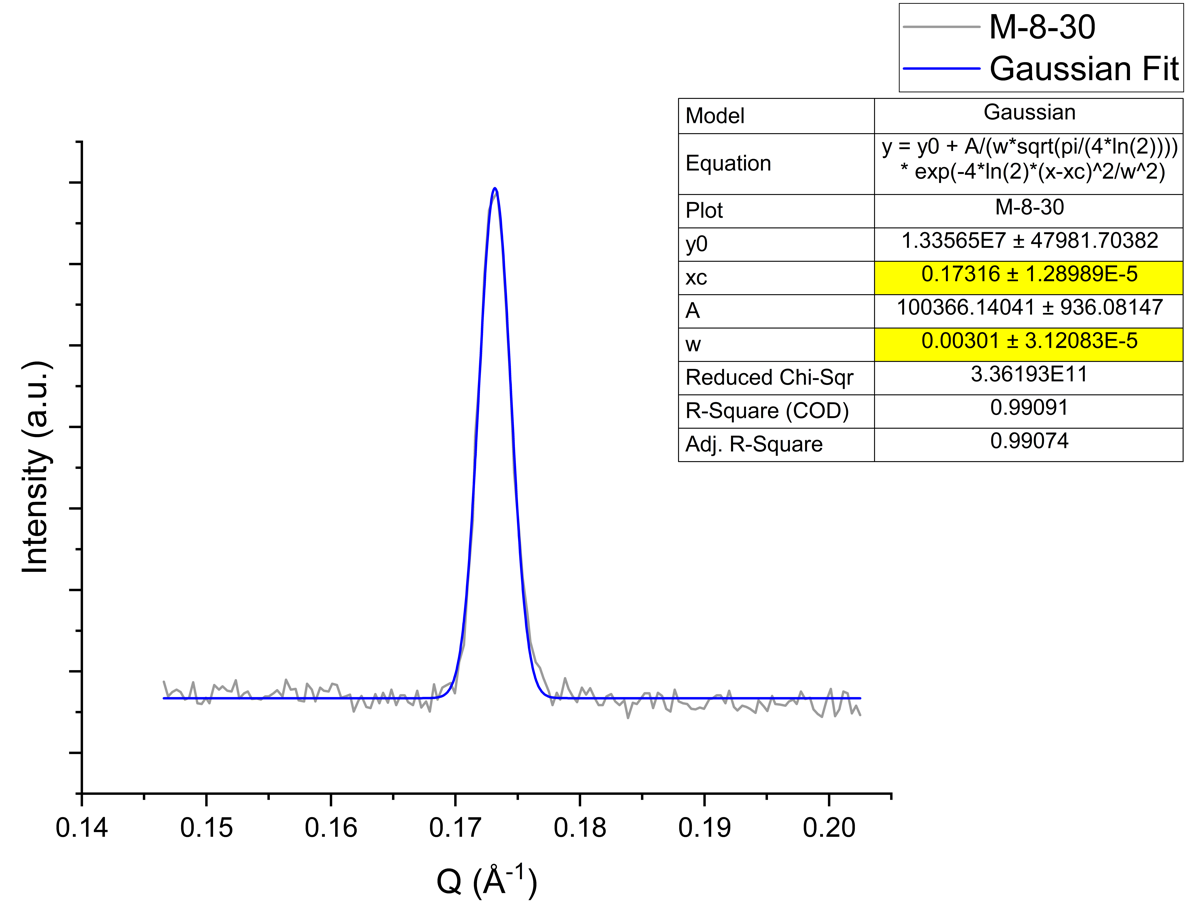
**

**Figure S7: Peak Fitting of SAXS**

**
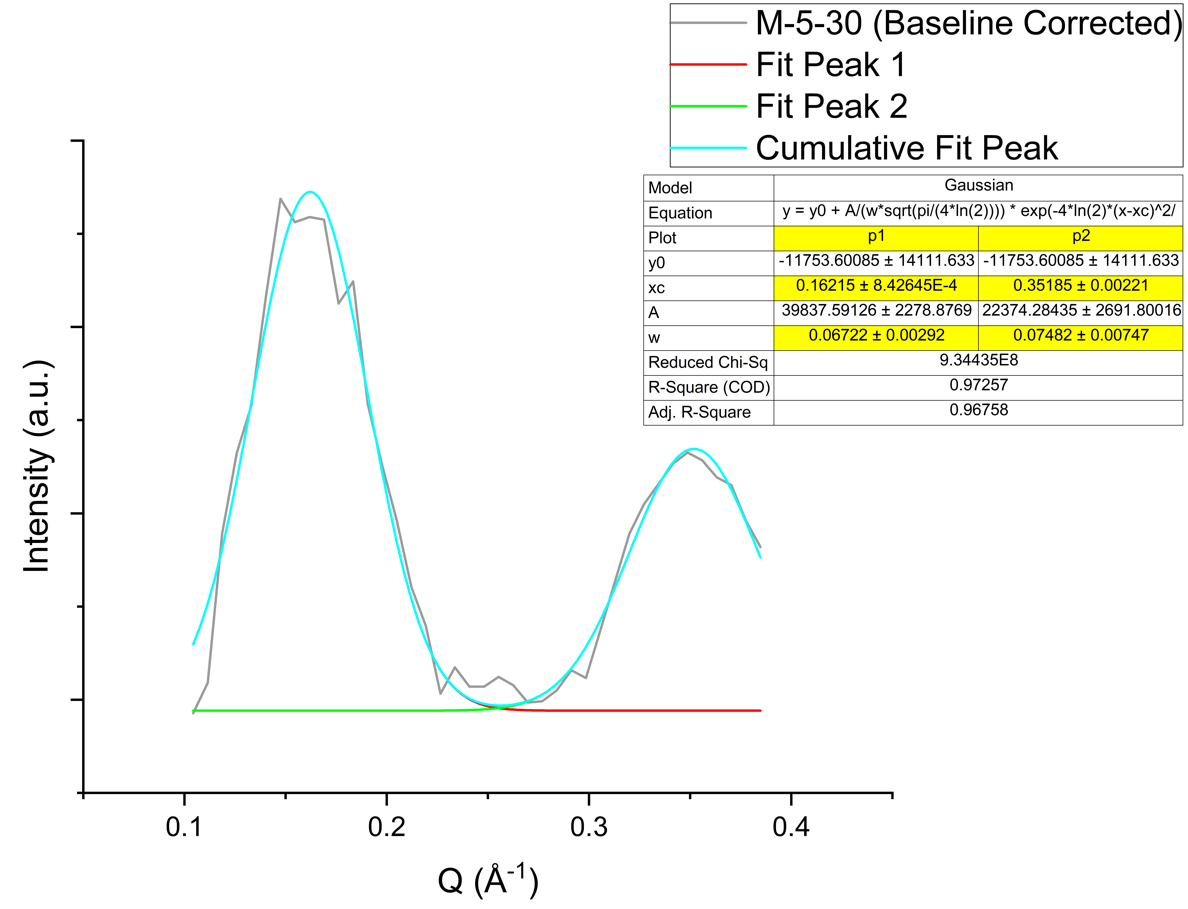

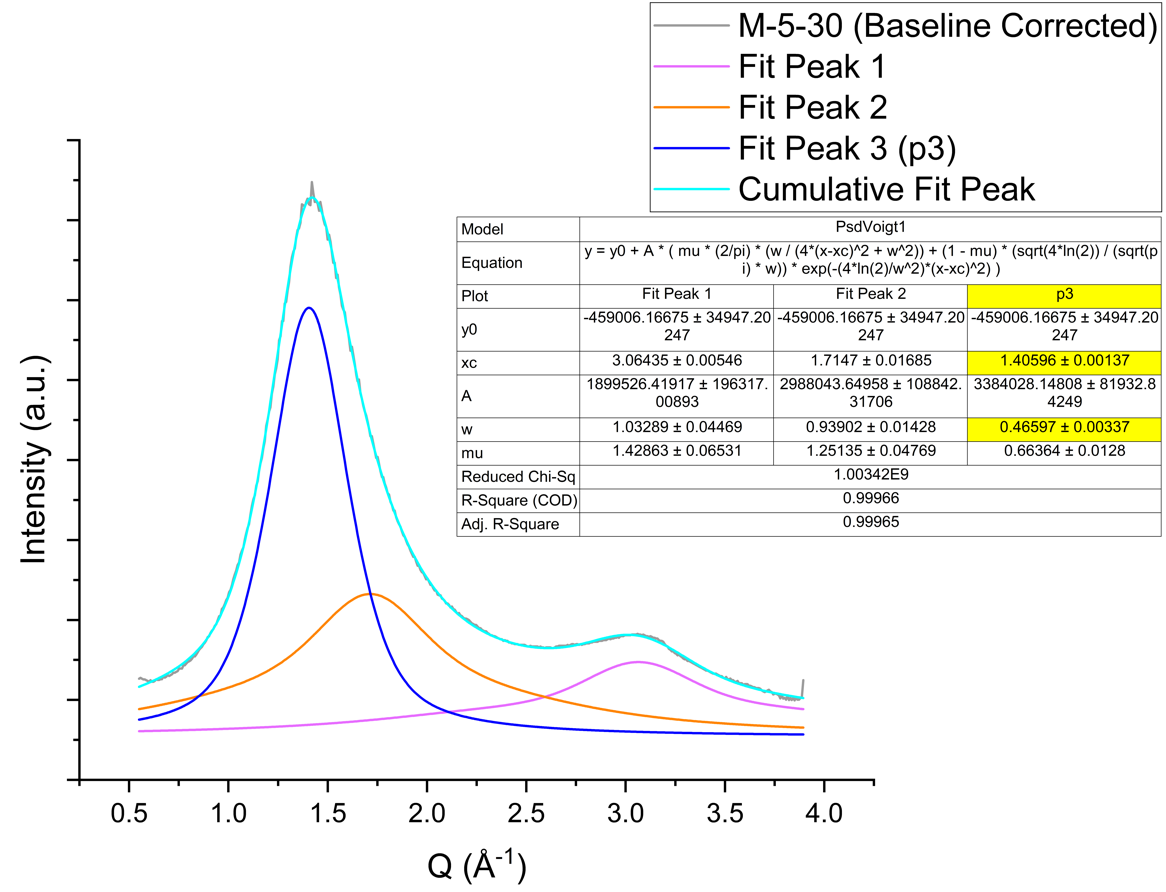
**

**Figure S8: Deconvolution and Peak Fitting of M-5-30 WAXS**

**
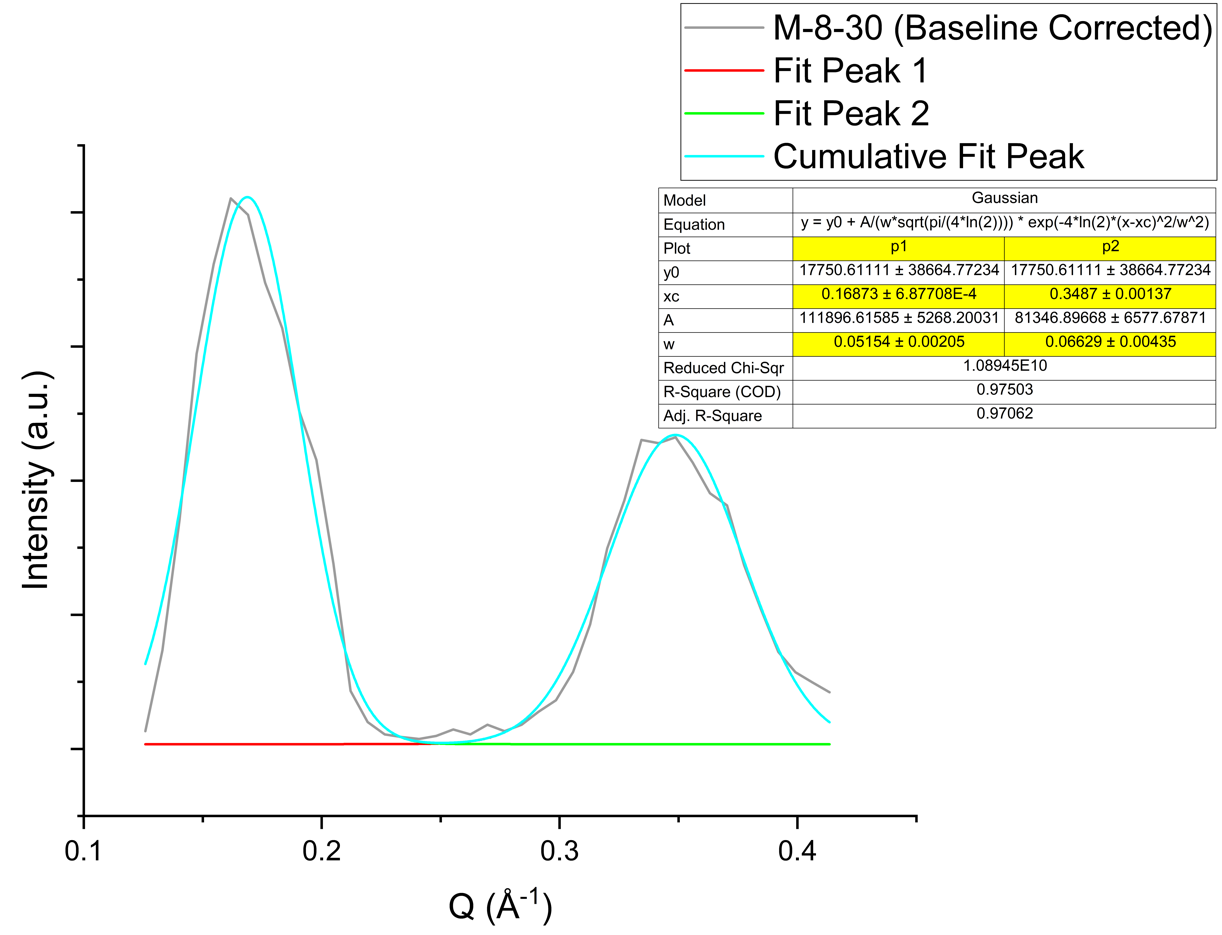

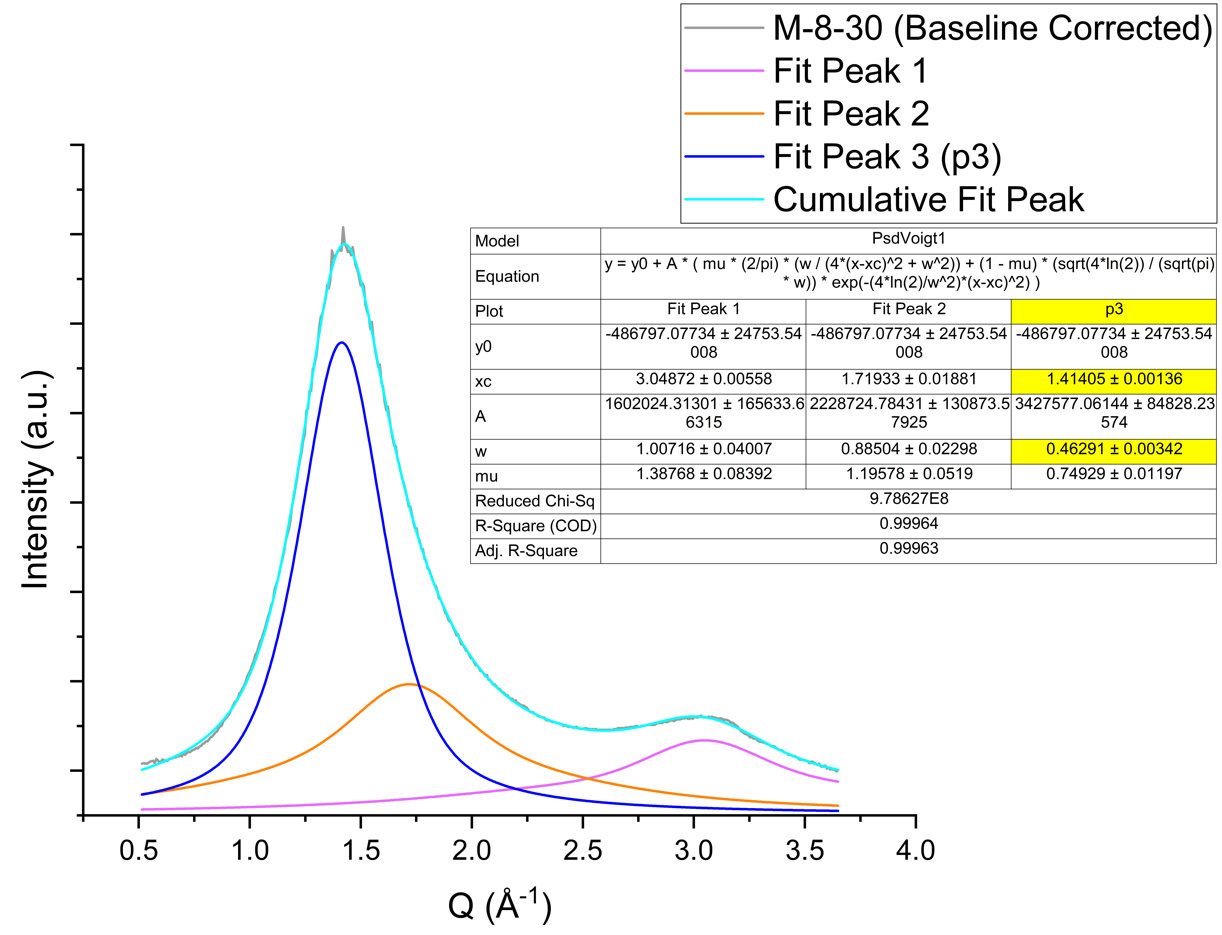
**

**Figure S9: Deconvolution and Peak Fitting of M-8-30 WAXS**

**Supplementary Note 6: Investigation of LC-LCE Time-Dependent Mechanical Properties**

Since polydomain thiol-acrylate LCEs are known to crystallize over time and contain unreacted acrylate ends that can thermally polymerize, with potentially an additional time-dependent effect of phase-separated domains being unknown, it was crucial to examine how the mechanical properties develop as a function of time. Polydomain samples were tested every 24 hours after synthesis over the course of 5 days, with n = 2 specimens of each sample tested on each day. Samples were heated above their T_NI_ on a hot plate and cooled to room temperature before testing to remove residual crystallization. A Linear Elastic Region (LER) develops over the course of 3 days for P-5-30 and P-8-30, and mechanical properties heighten on day 5 of the experiments. It is tempting to attribute this to the growth of phase-separated domains. But we did not have sufficient evidence to do so. A potential systematic study to answer this question would involve monitoring the size of such domains over time for every single composition. Moreover, characterization methods such as time-dependent SAXS/WAXS and TEM, and implementing them to all compositions, would be instrumental for such investigations. At the moment, a systematic investigation of this nature is out of the capabilities of our group.


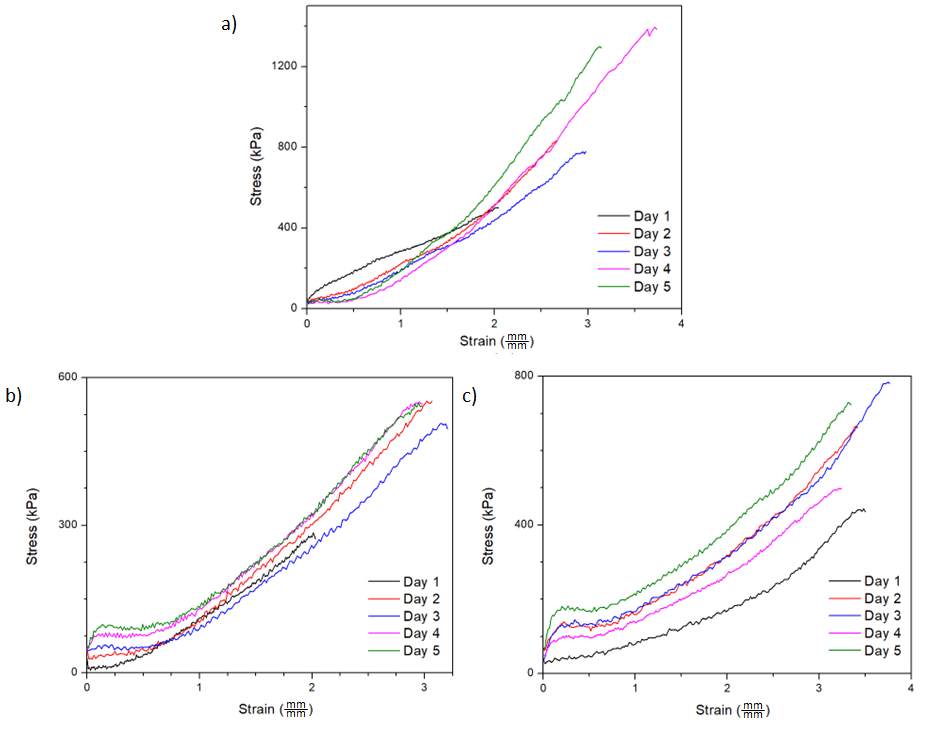


**Figure S10: Time-Dependency of LER observed in LC-LCEs.** a) P0, b) P-5-30, c) P-8-30

All formulations show the development of a soft-elastic plateau (SEP) and strain hardening, with the steepest strain hardening recorded on Day 5. As more LMWLC is introduced, the SEP constitutes a larger percentage of the whole stress-strain curve. Strain hardening is associated with polymer chains being sufficiently uncoiled, wherein more load is required for further deformation. Day 5 is also when P-5-30 and P-8-30 display the highest SEP, indicative of a larger threshold stress being required for uniaxial elongation with no additional stress. Intriguingly, a linear elastic region (LER) evolves by Day 2 in P-8-30 and Day 3 in P-5-30, which maximizes by Day 5, indicating a markedly larger resistance to initial loading. Bearing in mind that LMWLC domains display cybotacticity, this resistance is related to a higher energy barrier for inciting rotation of these short-range smectic domains.

**Supplementary Note 7: Estimating the Elastocapillary Length**

To estimate the elastocapillary length, we had to measure the surface tension of LMWLCs within a medium surrounded by LCEs. Theoretically, the best way to do this is to measure the contact angle of a pendant droplet of LMWLC within LCE precursors. However, this is a very challenging procedure as the LCE precursor is highly prone to polymerization, even at room temperature, very viscous, and optically very similar to LMWLCs. As such, we crudely estimated the surface tension between LMWLC and LCEs from the measurement of the contact angle of 5CB on LCEs (Figure S1). The interfacial energy of the LCE with LMWLCs was studied by determining the surface energy of P0. First, contact angles of deionized water, ethylene glycol, dimethylsulfoxide, and formamide with the LCE were measured using a goniometer. Then, P0's surface energy was calculated with the Owens-Wendt method ^[10]^. This was calculated as 30.7mN/m. The interfacial energy between P0 and 5CB was calculated as 2.12 mN/m with Young's equation, using 5CB's measured contact angle with the LCE (35.5°) and 5CB's surface energy at 25°C (35.1mN/m) ^[11]^. The interfacial energy between P0 and 8CB was not studied since 8CB was too viscous at ambient temperatures (22-25°C) to flow through the goniometer needle, and the goniometer setup did not allow for heating 8CB to decrease its viscosity. However, considering the similarity between P0's surface energy and 8CB's surface energy at 25°C (29.6mN/m), it is expected that their interfacial energy is quite small (i.e., below 10mN/m). Upper and lower limits of elastocapillary length can then be calculated as $L\approx\gamma/E$. For the lower limit, we assume the interfacial tension to be 2.12 mN/m and for the upper limit, we assume it is equal to the surface tension of the solid LCE (30.7mN/m). Taking $E_{P0}<30 kPa$, $71 nm<L_{P0}<1000 nm$. Taking $E_{M0}<3 MPa$, $0.71 nm<L_{M0}<10 nm$. Note that these values are only crude estimates of elastocapillary length limits. In our TEM experiments, we identified large populations of phase-separated domains with characteristic radii ranging from 30 nm to 600nm, which fit well within the approximated elastocapillary limits.

**Supplementary Note 8: Estimating the Isotonic Output Work Density**

While the enhanced stiffness of LC-LCEs may suggest their efficient use in artificial muscle applications, it is only one of many properties that can be related to muscular performance. The ability of the LCEs to control and direct loads is intrinsically relevant to this purpose. Using dead weight, observing the initial strain under load and active thermal stroke while the LCEs are under load, and subsequently calculating the output work density, parallels the human muscle’s eccentric and concentric contractions, respectively, and reflects the muscle’s ability to do work. Below in Table S5, these observations and calculations are summarized, and a photograph of the testing apparatus is detailed in Figure S11.

Lower stretched length values indicate strong eccentric movement, the ability to resist and control loads. As this is directly related to stiffness, it is no surprise that M-5-30 and M-8-30 do not initially deform as much as M0. For the active stroke, derived from the final strain and initial strain, somewhat higher values are observed for LC-LCEs and suggest their superior ability to move loads. Ultimately, the output work density for LC-LCEs significantly outperforms that of the control LCE. This is not only on account of their higher active thermal stroke, but also their light weight due to their smaller solid fraction.

Therefore, the stiffness enhancement of LC-LCEs extends to their ability to do muscle-like work. Their superior ability to resist loads, move loads over longer strokes, and lightweight character are desirable for artificial muscles. Note that the larger work density of M-5-30 than that of M-8-30 can be attributed to the inherently time-dependent and batch-to-batch variability in the synthesis of LC-LCEs. The bottom line is at least a 2-fold enhancement of the work density of LCEs when they are doped with LMWLCs.

**Table S5: Summary of Active Thermal Stroke Testing Data**

| Sample | Initial Gauge Length (mm) | Stretched Length (mm) | Final Length (mm) | Initial Strain  (%) | Active Thermal Stroke (%) | Average Sample mass (mg) | Dead weight (g) | Output Work Density (J/kg) |
| --- | --- | --- | --- | --- | --- | --- | --- | --- |
| M0 | 5.0 | 5.9 ± 0 | 5.2 ± 0.15 | 18 | 14.5 ± 3.5 | 58 | 75 | 9.5 ± 1.9 |
| M-5-30 | 5.0 | 5.2 ± 0.1 | 4.0 ± 0.1 | 4 | 28.5 ± 6.4 | 34 | 75 | 25.2 ± 0.4 |
| M-8-30 | 5.0 | 5.5 ± 0.25 | 4.6 ± 0.2 | 10 | 18.5 ± 0.7 | 35 | 75 | 18.1 ± 1.0 |


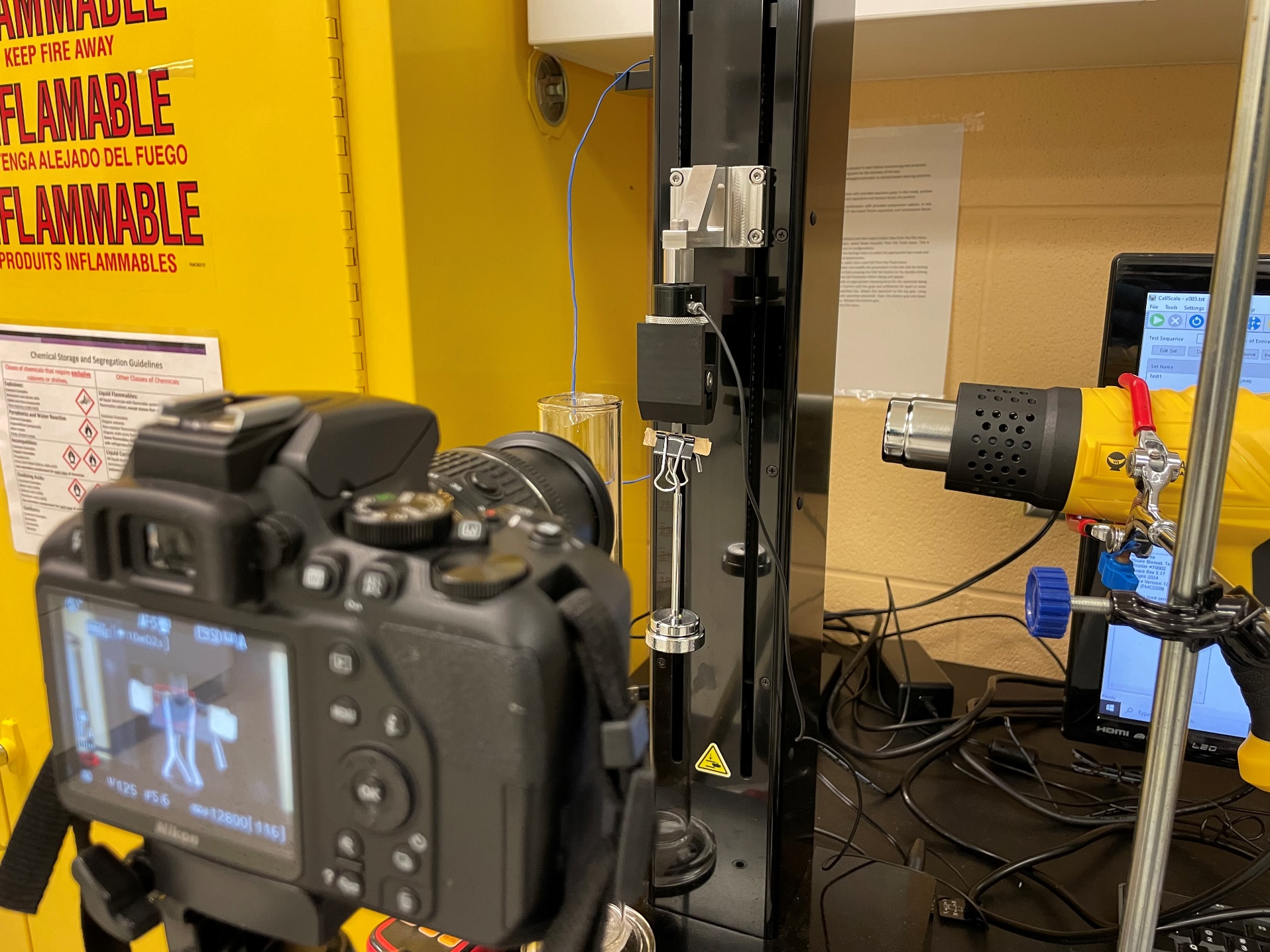


**
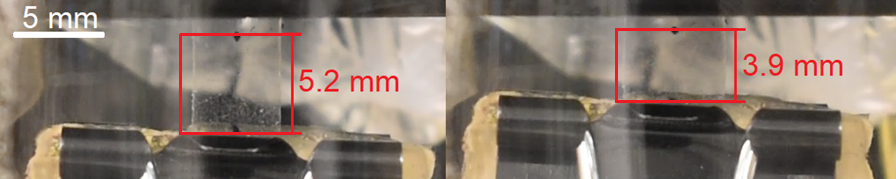
**

**Figure S11: Performing the Active Thermal Stroke**

**Supplementary References**

[1] R. W. Style, R. Boltyanskiy, B. Allen, K. E. Jensen, H. P. Foote, J. S. Wettlaufer, E. R. Dufresne, *Nat Phys* **2015**, *11*, 82.

[2] N. Bouchikhi, F. Semdani, L. A. Bedjaoui, U. Maschke, *Molecular Crystals and Liquid Crystals* **2012**, *560*, 159.

[3] N. Bouchikhi, L. Alachaher‐Bedjaoui, T. Bouchaour, G. ‐J. F. Tabieguia, U. Maschke, *Macromol Symp* **2014**, *336*, 68.

[4] H. Shahsavan, A. Aghakhani, H. Zeng, Y. Guo, Z. S. Davidson, A. Priimagi, M. Sitti, *Proceedings of the National Academy of Sciences* **2020**, *117*, 5125.

[5] Y. Yusuf, Y. Ono, Y. Sumisaki, P. E. Cladis, H. R. Brand, H. Finkelmann, S. Kai, *Phys Rev E* **2004**, *69*, 021710.

[6] P. A. Small, *Journal of Applied Chemistry* **1953**, *3*, 71.

[7] C. Luo, C. Chung, C. M. Yakacki, K. Long, K. Yu, *ACS Appl Mater Interfaces* **2022**, *14*, 1961.

[8] L. Yu, H. Shahsavan, G. Rivers, C. Zhang, P. Si, B. Zhao, *Adv Funct Mater* **2018**, *28*, 1802809.

[9] H. Guo, M. O. Saed, E. M. Terentjev, *Adv Funct Mater* **2023**, *33*, DOI 10.1002/adfm.202214918.

[10] D. K. Owens, R. C. Wendt, *J Appl Polym Sci* **1969**, *13*, 1741.

[11] M. Tintaru, R. Moldovan, T. Beica, S. Frunza, *Liq Cryst* **2001**, *28*, 793.
